# Reconstruction of an extinct soundscape reveals ultrasonic communication in the Jurassic

**DOI:** 10.1073/pnas.2615107123

**Published:** 2026-08-25

**Authors:** Jun-Jie Gu, Fernando Montealegre-Z, Thorin Jonsson, Charlie Woodrow, Emine Celiker, Md Niamul Islam, Jackson B. Linde, Fabio A. Sarria-S, Fuming Shi, Hojun Song, Daniel Robert, Dong Ren

**Affiliations:** ^a^ College of Agronomy, Sichuan Agricultural University, Chengdu 61130, Sichuan, China; ^b^ College of Life Sciences, Capital Normal University, Beijing 100048, China; ^c^ School of Natural Sciences, Joseph Banks Laboratories, University of Lincoln, Lincoln LN6 7DL, United Kingdom; ^d^Institute of Biology, Karl-Franzens-University Graz, Graz 8010, Austria; ^e^ School of Engineering, University of Leicester, Leicester LE1 7RH, United Kingdom; ^f^ School of Life Sciences, Arizona State University, Tempe, AZ 85287; ^g^ College of Life Sciences, Hebei University, Baoding, Hebei Province 071002, China; ^h^ School of Biological Sciences, University of Bristol, Bristol BS8 1TQ, United Kingdom

**Keywords:** extinct insects, paleoacoustics, Laser Doppler Vibrometry, Phlylogenomics, wing vibrations

## Abstract

Very little is known about the acoustic landscape of long-gone environments, such as the Jurassic forests. The sounds made by dinosaurs and other charismatic vertebrates are not definitively known because their vocal organs rarely preserve well in fossils. Unlike tetrapod vocal cords, the sound-producing organs present in the sclerotized cuticle of some arthropods do fossilize well. For example, the stridulatory structures (file, plectrum) can be observed and measured in the fossilized forewings of male crickets and allies. These fossils incorporate a fingerprint of the acoustic signals they generated, offering a unique window into the soundscapes of the past. While call frequencies can be inferred from fossilized wings using phylogenetically informed predictions, the reconstruction of wing vibrations and the songs’ temporal patterns from fossil material remains elusive. Integrating phylogenetics, laser Doppler-vibrometry, numerical simulations, and an AI-based approach, 20 ensiferan fossils (nine species) from a single location (Jiulongshan Formation, Inner Mongolia, China) were studied to recreate the acoustic landscape of the Middle Jurassic. These insects produced pure-tone calls, an adaptation to avoid localization by eavesdropping predators. They also exhibited rich diversity in their calling song frequencies and repertoires, facilitated by specialized file morphologies and wing-size variation. One species called above 20 kHz, suggesting that ultrasonic communication in insects was established long before the emergence of bats in the Eocene. Evolving mammalian hearing thresholds suggest early predators imposed acoustic pressures before bats. This arms race with singing ensiferans likely drove Jurassic insect song diversity and the evolution of mammalian and insect auditory systems.

## Significance

The acoustic environments of ancient ecosystems such as Jurassic forests are poorly known. While the vocal organs of dinosaurs and other vertebrates rarely fossilize, the sound producing structures in the sclerotized cuticle of some arthropods do. Stridulatory files and plectra preserved in the fossilized wings of katydid ancestors retain measurable features that reveal aspects of their calls. Using phylogenetics, laser Doppler vibrometry, numerical simulations, and an AI-based approach, we analyzed 20 fossils from Inner Mongolia, China. We reconstructed their acoustic landscape, showing pure tone, diverse, and in some cases ultrasonic calls, with implications for early insect–mammal coevolution.

A salient feature of biodiverse habitats is the acoustic landscape, which reveals key animal interactions and their ecologies. From dense tropical forests to tundra and coral reefs, natural habitats are teeming with biologically generated acoustic signals, produced by animals for intra- and interspecific communication (1). These soundscapes and their individual generators are easily heard and recorded by the present-day observer, but harder to establish for environments of the past. Undoubtedly, the forests, grasslands, and steppes of the Cenozoic and Mesozoic were filled with acoustic signals emitted by a rich diversity of both invertebrates and vertebrates (2–4). While sound itself does not fossilize, we can infer the sounds of the past by either hypothesizing the ancestral sound-producing and hearing traits through ancestral state reconstruction based on the traits of extant group (5–7), or use the fossilized remains of sound-production and sound receiving organs to indirectly draw conclusions on the ancient soundscapes using predictions and biophysical models (3, 8). However, this latter approach is often limited because key vocalization organs are membranous, cartilaginous, or otherwise soft tissue, and therefore rarely preserve. Examples of such fragile organs include the resonating air sacs in frogs, the larynx and vocal cords in mammals, the syrinx in birds, and the sound box in dinosaurs (9). Evidence of sound-producing soft tissue in the fossil record is rare and hardly extends beyond the Cenozoic (4, 9, 10). A notable exception is a Late Cretaceous avian syrinx (66-69 ma) belonging to a member of the Anseriformes (waterfowl), which was likely able to produce duck- or goose-like honking sounds (11), and the larynx in a nonavian tetrapod, a pre-Cenozoic *Pinacosaurus*, which is believed to have produced loud booming calls as in many reptiles and birds (9). Other examples of fossilized acoustic structures are the osseous cranial crests of Late Cretaceous hadrosaurs (Ornithischia, Hadrosauridae) that are hypothesized to have functioned as resonance chambers for low-frequency social communication signals (12).

In insects, acoustic communication is widespread and has evolved independently multiple times (13) since probably the Early Permian (3, 4). Crickets, katydids (bush-crickets), and their allies (Orthoptera, Ensifera) are dominant contributors to our planet’s soundscapes, and have diversified since their emergence in the Late Carboniferous (7). Most extant male katydids (Orthoptera, superfamilies Tettigonioidea and Hagloidea) produce loud calling songs by wing stridulation to attract distant females. The passing of a serrated wing vein (the stridulatory file) on one wing over a plectrum on the opposite wing produces sounds that are further enhanced and radiated by specialized vibrating wing regions (14, 15). The wings open and close rhythmically, but sound is mainly produced during the closing phase (*SI Appendix*, Figs. S1–S4 and Movie S1). A single closing phase produces the most basic call unit, termed here a “syllable.”

**Fig. 1.**
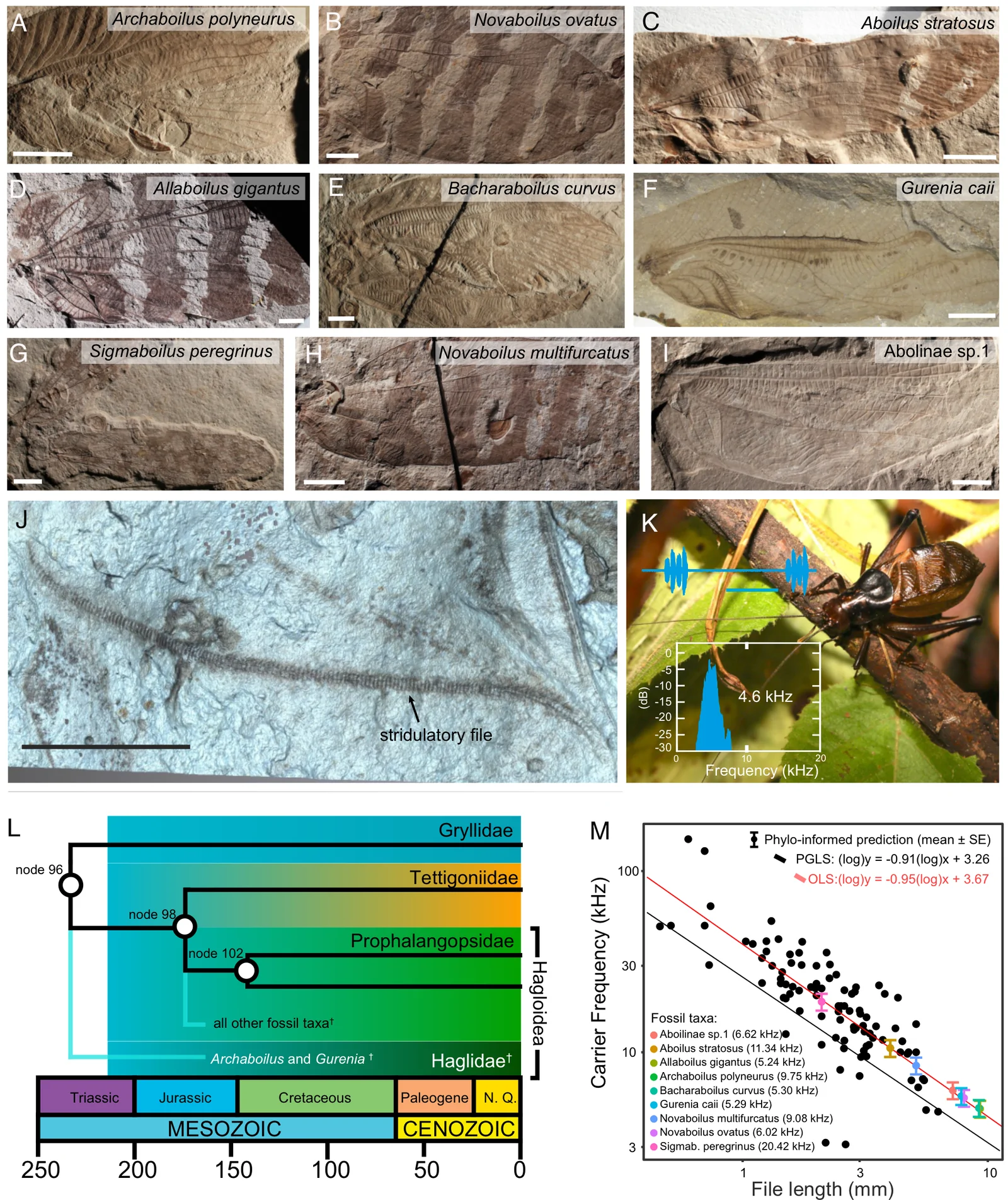
Fossilized wings and phylogenetically informed predictions of carrier frequency. (*A–I*) Examples of fossilized wings of the nine species presented in this study. (*J*) View of the stridulatory file of *Bacharaboilus curvus* (Prophalangopsidae). (*K*) *Tarragoilus diuturnus* (a living member of the Prophalangopsidae) displaying call oscillogram and acoustic spectral energy. (*L*) Broadest phylogenetic position of fossils used in this study, and indication of nodes between which taxa are positioned based on taxonomy. (*M*) Regression models and phylogenetically informed predictions of carrier frequency from file length for 95 extant ensiferans (black dots), and predictions for fossil taxa based on mean file lengths for each species (see symbols for each species). The red line indicates non-phylogenetically informed correlation (OLS model), the while black line indicates the phylogenetically controlled PGLS model. Colored points and error bars represent phylogenetically adjusted predictions from the PGLS model. (Scale bar, *A*–*L* = 5.0 mm; *J* = 2 mm; *K* = 0.2 s.)

**Fig. 2.**
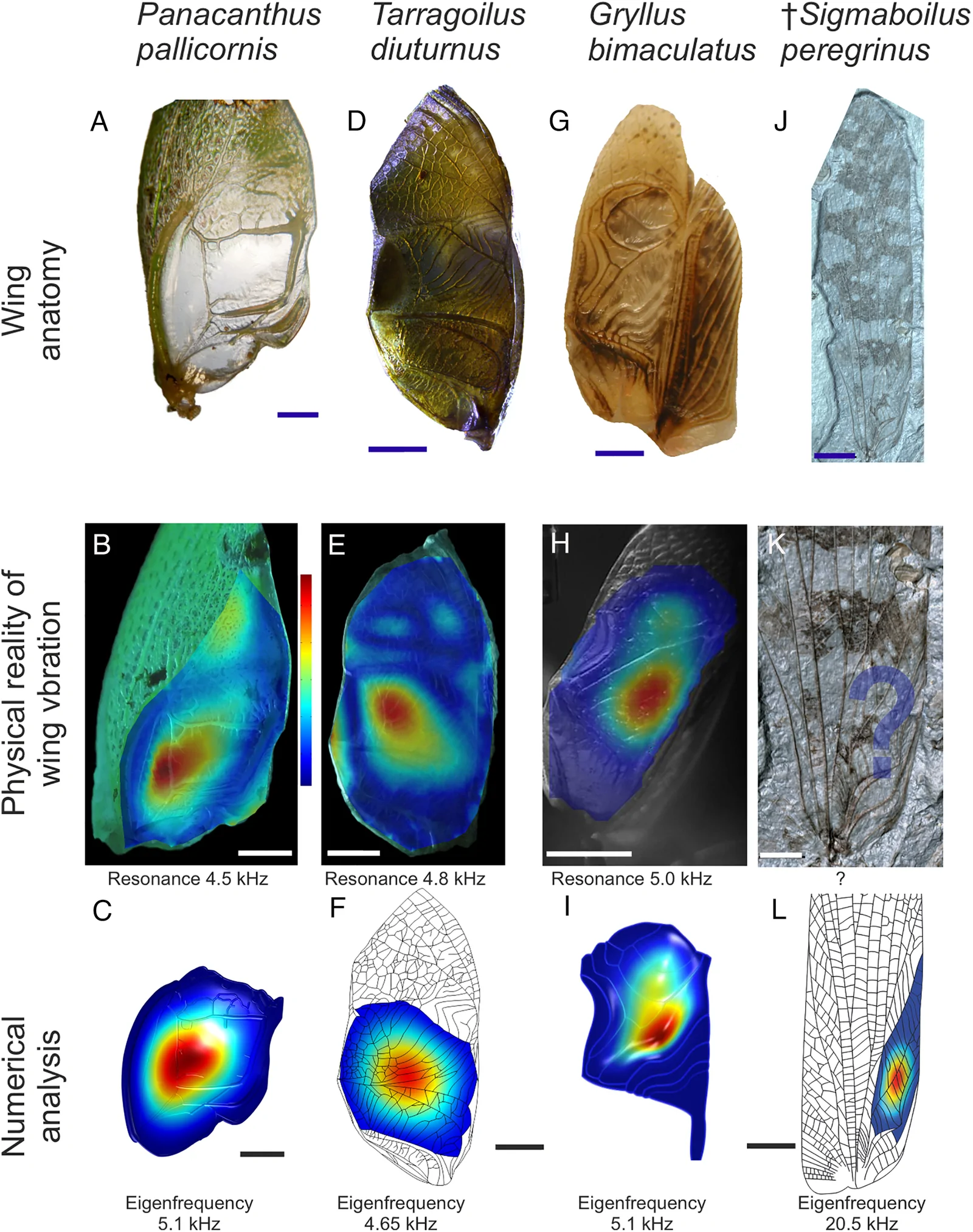
Validation of FEA models for extracting wing vibrations from fossilized wings, using living species as proof of concept. Morphology (*top row*), LDV experimental data (*middle row*), and FEA results (*lower row*) of three extant and one extinct Ensifera. (*A–C*) *Panacanthus pallicornis* (LDV at 4.4 kHz, FEA at 5.0 kHz, songs 4.6 to 6.1 kHz, f at ~5 kHz). (*D–F*) *Tarragoilus diuturnus* (LDV at 4.4 kHz, FEA at 4.6 kHz, songs 4 to 5 kHz). (*G–I*) *Gryllus bimaculatus* (LDV at 4.9 kHz, FEA at 5.1 kHz, songs 4.8 kHz). (*J–L*) *Sigmaboilus peregrinus* (physical reality not possible to assess via LDV), FEA 20.50 kHz, *f*_c_ estimated at 20.42 kHz. (Scale bar, 3 mm.)

In katydids, crickets, and grigs, the carrier frequency (*f_c_*) of the mating calls matches the natural vibration of the wings (16, 17). For example, to optimally produce a *f_c_* of 5 kHz, the wings should resonate best at 5 kHz, and they should close with a velocity that drives the plectrum over the file engaging at a tooth strike rate of 5,000 teeth per second (18, 19) (Movie S1). Thus, as acoustic radiators, wings should be structured so that, when stimulated at their natural frequencies, they vibrate easily and therefore radiate acoustic energy at optimal amplitudes. Exploiting natural resonances allows loud sound production at low energetic cost (20, 21). Resonances in ensiferan wings depend on wing size, dimensions of substructures involved, and other physical parameters (20, 22). Many katydids generate pure-tone signals with highly resonant wings, while others create broadband songs with broadly tuned or nonresonant wings (23). The frequencies exploited across species range from ~1 to 150 kHz, with ca. 70% of known extant species using ultrasounds (i.e., sounds above the human hearing limit of ~20 kHz) (22). The chitinous exoskeleton of crickets, katydids, and allies easily fossilizes, and so do their sound-producing organs (the wings), which can be used to infer the nature of signals generated, as wing anatomy is a reliable predictor of acoustic features of their calls (3, 24–26). To investigate stridulatory sound production in Jurassic relic Ensifera, we can turn to their closest living relatives: the relict family Prophalangopsidae (e.g., *Tarragoilus diuturnus*, *Cyphoderris* spp., and *Prophalangopsis obscura*). These “living fossils” retain wing morphologies and stridulatory behaviors that are putatively deeply conserved, preserving key ancestral acoustic features. As a result, these insects provide a rare, functionally informed window into how early katydids—and their Jurassic ancestors—may have generated sound (17, 27). Notably, the sound generators of the living Prophalangopsidae (also known as grigs) look more like their Jurassic ancestors than the extant katydids (Figs. 1 and 2 *D*–*I*). There are approximately 100 described species of Prophalangopsidae, of which only nine extant species still exist in temperate habitats across China, India, Russia, the United States, and Canada, while the remainder are known only from the fossil record (28).

Wing stridulation in the katydids’ ancestors (stem-Ensifera) is known from the Late Permian and Middle Triassic (29–31) and was already well-established and diversified by the Late Triassic and Middle Jurassic (174 to 163 ma) (3, 25). It is known that, ~165 ma, *Archaboilus musicus*, a member of the extinct Haglidae (a family closely related to Prophalangopsidae and Tettigoniidae), produced calling songs by stridulation with low-frequency tones at ca. 5 kHz (22, 25, 32). In contrast, katydids (Tettigoniidae) are not known in the fossil record before the Cenozoic (24, 26), although recent molecular data suggest that the Tettigoniidae originated in the Late Cretaceous (7), but no fossils are available for that time. Two species of Tettigoniidae from the Middle Eocene (~55 ma) with intact stridulatory organs, *Pseudotettigonia amoena* and *P. leona*^4-5^, have enabled the inference of a *f*_c_ of 10.5 and 11 kHz, respectively, using phylogenetic comparative methods (22), and one species of katydid in amber from the Eocene has allowed for the reconstruction of both acoustic signaling and hearing at 32 kHz (33). These examples demonstrate the use of several communication channels by extinct insect species, at least through variation in song frequency, and the suitability of the Ensifera as a model for the reconstruction of the extinct soundscape.

As the shift from low- to high-frequency signaling and the proportional hearing abilities in katydids appears broadly contemporaneous with the rise of bats (33–36), it is tempting to interpret their ultrasonic communication as an adaptation to bat predation. This adaptation could be through a requirement to detect echolocating bat ultrasound, making a shift to ultrasonic signaling a low-cost strategy for hearing both predators and conspecifics; or through the fact that ultrasounds fade rapidly in an environment, so a distant eavesdropping bat may struggle to localize individuals. Yet, this timing does not uniquely implicate bats: early bats were themselves sensitive to high frequencies, and bat stem lineages may have possessed some capacity for echolocation (37). Moreover, high-frequency hearing is thought to have been present in nocturnal mammalian ancestors long before bat echolocation evolved (6, 38). Taken together, these observations motivate three, not mutually exclusive, hypotheses for the origin of ultrasonic signaling in katydids: i) ultrasonic communication evolved in response to eavesdropping by bats (ultrasounds do not travel far, so it is hard for a predator to localize distant prey); ii) ultrasonic traits arose prior the evolution of bats as a general anti-eavesdropping strategy against earlier predators with high-frequency hearing sensitivity; and iii) ultrasound evolved through acoustic niche partitioning, through changes in wing structure and body size, allowing signaling to escape masking and interference in increasingly noisy communities of sympatric callers (39).

To test these hypotheses, we used a combination of phylogenetic regressions and informed predictions of extant species, biomechanical experimentation on living forms, and numerical analysis, to reconstruct wing vibrations and acoustic signals of 20 fossil ensiferans from the Middle Jurassic (~165 ma; consisting of seven species within the Prophalangopsidae and two within Haglidae—an extinct family that existed from the Triassic to Cretaceous). All 20 fossil specimens offered exceptionally well-preserved stridulatory structures (Fig. 1 *A*–*J* and *SI Appendix*, Fig. S5) and originated from the Jiulongshan Formation of Daohugou village (Nei Mongol Autonomous Region, China). Because these species co-occurred in both time and place (157 to 165 Ma) (40), they form a true paleo-acoustic assemblage—the oldest yet assembled. This provides an ensemble framework for paleo-acoustic inference, enabling the reconstruction of a substantial portion of the Middle Jurassic soundscape with unprecedented detail.

## Results

### Biomechanics of Living Wings vs. the Unknown Biomechanics of Fossilized Wings.

#### Wing stridulation biomechanics and sound radiation in living forms.

We examined living species from three families (Prophalangopsidae, Gryllidae, and Tettigoniidae) with well-characterized calling songs. Using micro-scanning laser Doppler Vibrometry (LDV), we measured wing resonance, damping, and vibrating areas, and captured wing mechanics with high-speed video (Movie S1) and motion detectors. As a proof of concept, using COMSOL multiphysics, we then built finite-element models of two-dimensional (2D) wing geometries, treating both call features and resonances as unknowns (Movie S2). 2D models are necessary to apply a similar and consistent process to fossilized wings, which can be recovered in 2D for modeling. The numerical models match the “physical reality” of the living wings, simply meaning the model accurately captures the real wing biomechanics (Fig. 2 *A*–*I*); we therefore validated the models. Hence, the same modeling approach used for living species could then be applied to fossilized wings, even though their physical reality is unknown.

#### Reconstruction of the fundamental syllable in living forms.

A syllable is made of continuous oscillations produced by individual but continuous tooth-strikes. We used the method proposed by Montealegre-Z and Mason (14), which recovers the basic oscillation of a single tooth strike from a real wing using LDV. Subsequently, a Matlab algorithm that uses a time vector, file teeth spacing, and the basic tooth-strike oscillation, was used to reconstruct the basic syllable amplitude envelope. The time and frequency components of this reconstructed syllable were compared with the original acoustic recordings (physical reality) of each living species. The reconstructed syllable matches the recorded pattern from singing males (*SI Appendix*, Figs. S1–S4). Again, this concept was used to reconstruct the fundamental syllable from fossilized wings.

#### Vibrations and eigenfrequencies in fossilized wings.

Understandably, the physical reality and acoustics of fossilized wings cannot be measured with LDV (Fig. 2 *J*–*L*). We therefore extracted wing geometries from these fossils (*SI Appendix*, Fig. S5) and created 2D models using Adobe Illustrator vector outlines, for further finite element analysis (FEA), implementing all the structural parameters used for extant species (Movie S3). Numerical simulations were conducted using the same approach described above for living insects. We used an eigenfrequency analysis to identify potential natural resonances of the vibrating area of the wings, based on the vibrating area observed in extant Prophalangopsidae (17) (Fig. 2*E*), and accepted the model when the natural frequency was in the range predicted by the phylogenetically informed model (next section, see Fig. 1). While this limits our model to the presented taxa as other ensiferans have unique wing morphologies that can affect wing vibration mechanics (41), it is sufficient for the species used in this study, and was used to allow us to simulate a vibration event in the wing membrane for syllable reconstruction (*SI Appendix, section D*).

### Determination of Song Frequency in Extant and Fossil Ensifera.

When comparing the stridulatory organs of extinct ensiferans (i.e., the forewings and the serrated file) with those of extant species, it becomes clear that the basic mechanism of song production in this group has not undergone significant changes since the late Triassic (3, 25) (Figs. 1 *A*–*J* and 4 *A* and *B* and *SI Appendix*, Figs. S1*A*, S2*A*, S3*A*, and S4*A*). To infer song frequencies in Ensifera, we first reconstructed a robust phylogeny of Tettigoniidea based on 147 extant taxa and 1,077 single-copy orthologs generated using target enrichment (42). We estimated divergence times based on this phylogenomic tree using a curated set of fossil calibrations. The resulting tree was trimmed down to 95 species for which calling songs and physical reality of wings were known (*SI Appendix*, Table S1). This tree was used for subsequent phylogenetic comparative methods. We generated ordinary least squares (OLS) and phylogenetic generalized least squares (PGLS) models to understand the relationship between file length and *f_c_* (Fig. 1*M*), building on existing models to develop an improved, phylogenetically informed approach to fossil song reconstruction (32). Rather than predicting fossil *f_c_* from file length simply using the PGLS model equation, we used a phylogenetically informed approach. We attached fossil taxa between two specified internal nodes in the phylogeny based on current taxonomy (Fig. 1) and computed the phylogenetic covariance between the fossil species and all species in the extant dataset. This allowed us to adjust the PGLS model predictions based on possible phylogenetic positions of the fossils. This was conducted across paired nodes that contain the fossil taxa based on taxonomy, to provide a predicted range of *f_c_* spanning the broadest possible phylogenetic positions.

The OLS model found a strong negative relationship between (log) file length and (log) *f_c_* (Fig. 1*M*, estimate = −0.95, t = −12.12, *P* < 0.001). The PGLS model also found a strong negative relationship between (log) file length and (log) *f_c_*, but with a slightly lower intercept and shallower slope (Fig. 1*M*, estimate = −0.91, t = −12.07, *P* < 0.001). Based on the PGLS model, we ran phylogenetically informed predictions of *f_c_* for each fossil species based on the mean length of the stridulatory file of each species. These predictions provided estimates and error ranges for *f_c_* (*SI Appendix*, Table S2). We concluded that the mean predicted *f_c_* values were our most conservative estimates and compared them with the results from computational modeling for syllable reconstruction.

**Fig. 3.**
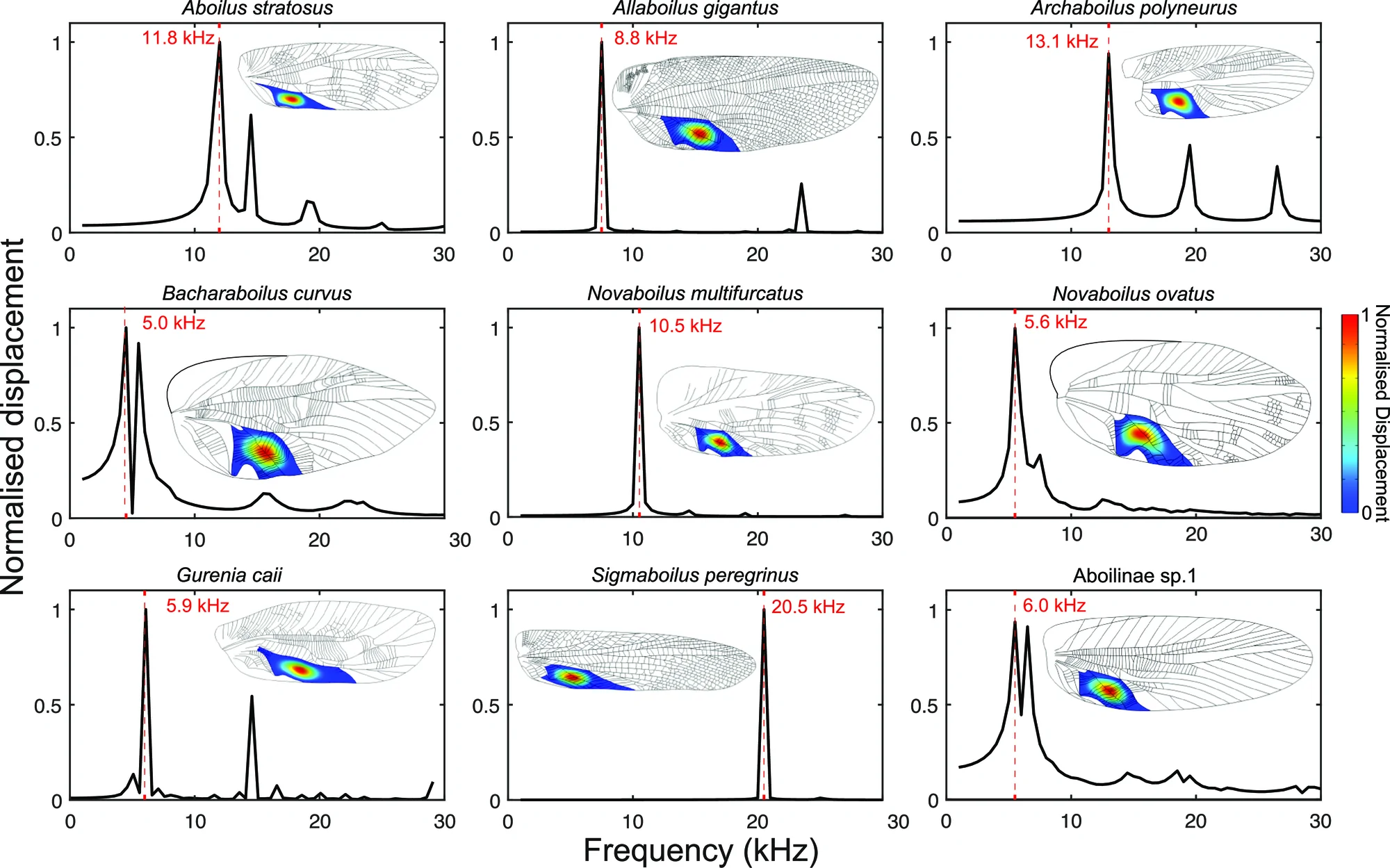
Reconstruction of main vibration of the stridulatory wing cells based on FEA models of nine fossil wing geometries. Deflection maps indicate relative minimum (blue) and maximum (red) deflection of the involved wing cells (see color scale). The quality factor *Q* (peak width at −3 dB) is measured from the fundamental frequency (dashed red line).

The results of these informed predictions indicate that Jurassic ensiferans already produced a great diversity of acoustic signals, from low frequencies around 5 kHz (e.g., *Bacharaboilus curvus*, *Novaboilus ovatus*, *Gurenia caii*) up to likely ultrasonic frequencies (*Sigmaboilus peregrinus*). These predictions overlapped well with the frequency estimates provided from numerical modeling (Fig. 3 and *SI Appendix*, Table S2), strengthening the support of our approach and confirming the likely acoustic signals produced by each species.

#### Reconstruction of fundamental syllable from fossilized wings.

**Fig. 4.**
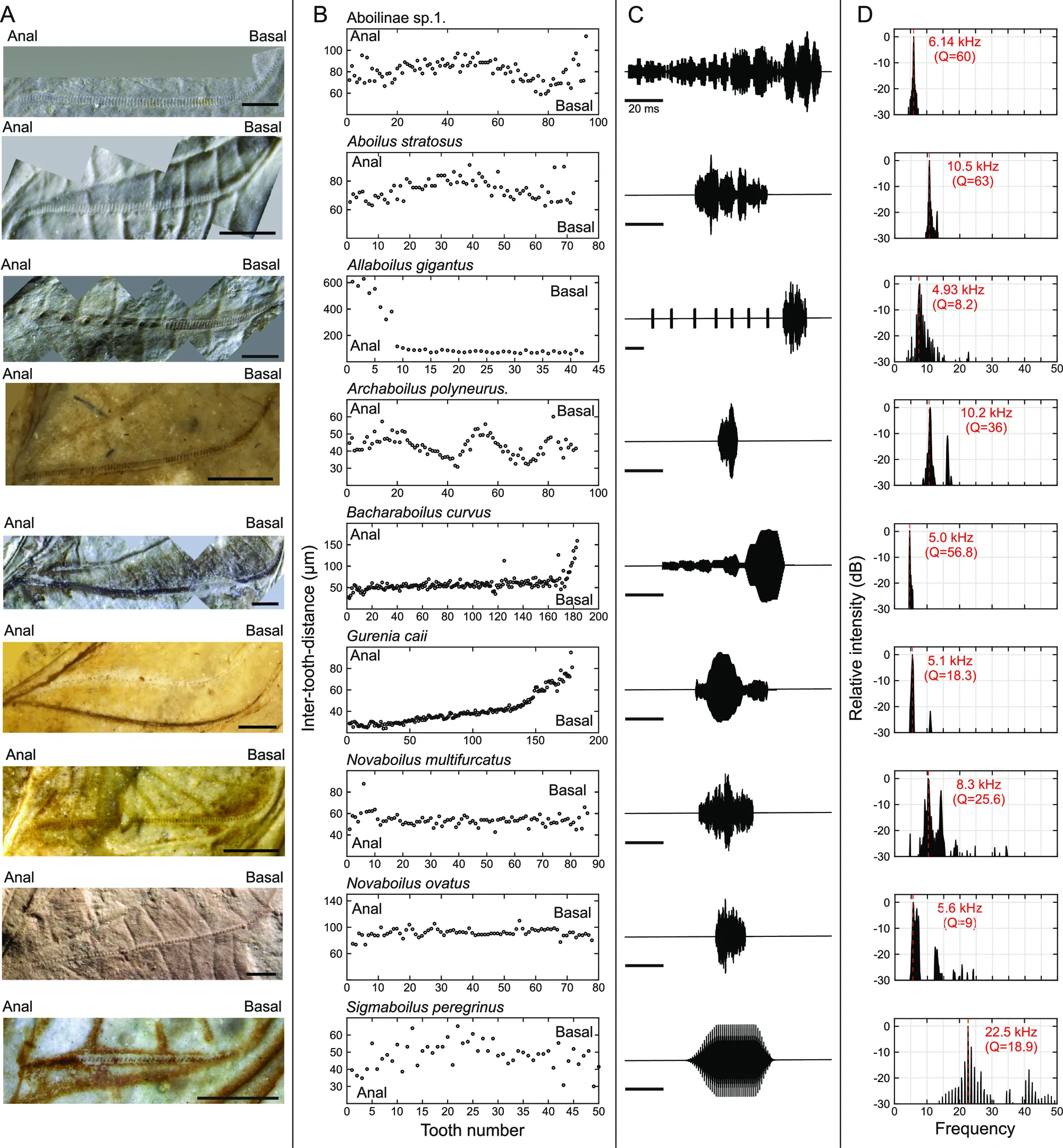
Reconstruction of the basic sound unit (syllable) of Jurassic ensiferan calls from stridulatory file morphology and model predicted carrier frequency. (*A*) A fossilized stridulatory file of each of the nine species. Orientation: *Top* is proximal, *Left* is anal. (Scale bar, 1 mm.) (*B*) Inter-tooth distances for each fossil file in *A*. Teeth number starts from first teeth on anal part of file. (*C*) Reconstruction of one syllable, based on tooth distribution, *f*_c_, and numerical models (see also Fig. 3). Oscillations are calculated as produced during the closing movement of the wings, i.e. the scraper would move from *Left* to *Right* in *A* and *B*. (Scale bar, 20 ms.) (*D*) FFT analysis of the reconstructed syllable. *f*_c_ shown in red.

Since fossilized wings do not allow us to produce file/plectrum clicks and record their vibrations using LDV (Fig. 2), we used the vibro-acoustic FEA models (Fig. 3) to recreate a single click and its basic oscillation pattern. For the time-dependent numerical simulations, a stridulatory file was excited with a force of 1.3 × 10^−3^ to 2.5 × 10^−3^ N, as inferred from high-speed videos of stridulating species (Movie S1). This caused the wing to enter resonance and vibrate in real time in response to the applied force. The resulting simulated vibrations of the wing were used to construct the fundamental syllable associated with each species, and the syllable was analyzed using FFT to confirm the vibration retained the expected *f*_c_ (see below and Fig. 4*C*).

These syllables represent the most basic and fundamental component of the call repertoire that can be recovered from fossilized wings. Other parts of the calls, like amplitude and frequency modulations and temporal patterns, are dictated by neural and behavioral variables (43) which do not fossilize, hence remain elusive in this analysis. Therefore, a Gaussian Process Regression (GPR) model was trained using the morphological and acoustic parameters of extant insect species to predict calling syllable rates of the fossilized insects. The model results demonstrated strong fit, low prediction error, and suitability for extrapolating to fossil species while maintaining biologically plausible uncertainty bounds. The predicted syllable rate was used to construct a hypothetical Jurassic soundscape (Movie S4 and Audio S1).

## Discussion

400 Mya, insects already occupied all terrestrial habitats (30) – long before their most notorious acoustically orienting predators, bats, who appeared in the Eocene (about 55 Mya) (34, 37). From pioneering work to recent days, it has been broadly accepted that the Orthoptera (grasshoppers, grigs, katydids, and crickets) are perhaps the first lineage of insects to use sound for conspecific communication (3, 25, 44). Airborne signaling by wing stridulation in ancient ensiferans was already established by the middle/late Triassic (3), and was honed over time with the evolution of sound generators evolved to produce pure-tone sounds. In extant katydid species, pure-tone sound production is associated with narrowly tuned wing resonators, and with files with regularly spaced teeth. In some species, such as some modern-day *Typophyllum* spp. (45)*, Peronura* spp. and *Melidia* spp. (46), the stridulatory files exhibit a distinctive and elaborate alignment of teeth, enabling tight control of the spectro-temporal composition of song (*SI Appendix*, Fig. S6).

Here, we show that sophisticated stridulatory files with structural mechanical features to produce pure-tone calls [e.g., *Liassophyllum caii*, *Bacharaboilus curvus*, *Aboilus stratosus*, see also *Archaboilus musicus* (25)] or to enhance bimodal temporal/frequency alterations (e.g., *Archaboilus polyneurus*, *Allaboilus gigantus*, Fig. 4), were already present and well developed in the Jurassic. Mechanical frequency modulation was thus already used by ancient ensiferans in the Jurassic. The three specimens of *Archaboilus polyneurus* show a regularly consistent pattern of increasing and decreasing intertooth distances (Fig. 4), consistent with the production of such amplitude and frequency modulations, as seen in a present-day pterochrozine leaf-mimicking katydids (45) (*SI Appendix*, Fig. S6). Similarly, the four examined specimens of *Allaboilus gigantus* exhibit an elaborated stridulatory file, which clearly shows two regions with different tooth densities (a constant-density region and one with irregularly spaced “pegs”; Fig. 4), which would produce different temporal and song frequency patterns. Analogous bipartite morphology can be observed in the files of some extant katydid species, such as the genera *Horatosphaga*, *Acrometopa, Tenerasphaga* (46), and the cricket *Eneoptera guyanensis* (47). This reveals that as early as the Jurassic, ancient ensiferans had evolved mechanical and structural wing designs that enhanced their song repertoire and facilitated *f_c_* diversification, and that these acoustic niches are still exploited today. What was the trigger of such early diversification?

The diversity of acoustic channels, and the pure-tone nature of these signals could have been driven by the need for acoustic niche partitioning in an increasingly noisy and species-rich environment, and also through the need to avoid the hearing range of eavesdropping predators [a form of evolutionary proactive antipredator behavior (48)].

Males of living species of Prophalangopsidae produce low-frequency pure-tones, exhibit strong wing symmetry, long stridulatory files, and some can produce sound during both wing opening and closing phases (17, 49) (*SI Appendix*, Fig. S1 *A*–*C*). The earliest diverging lineage of Tettigoniidae also exhibits wing symmetry (50). Such wing symmetry is also found in the fossils studied here, implying that the ancestral condition for sound production involved the main amplitude components of the call produced during both the opening and closing phases of the wing stridulatory cycle. Five of the nine species studied here used low-frequency pure tones (5 to 7 kHz), and from previous work on *Archaboilus musicus* (22, 25, 32) (using pure tones at ~5 to 6 kHz), it is evident that low-frequency pure-tones have been used as communication signals since Jurassic times, and still are in field crickets (Gryllidae) and in some Prophalangopsidae today. It is well known that pure-tones at around 5 kHz are less affected by spatial sound attenuation when signaling close to the ground, than higher frequencies (51, 52). Other species singing above 10 kHz (like *Aboilus stratosus*, *Novaboilus multifurcatus,* or *Sigmaboilus peregrinus*; Fig. 4) could have started to find more favorable niches for sound transmission above ground, like understory vegetation or more arboreal perches, as we see today in *Cyphoderris* (53) and *Paracyphoderris* (54), or even in many katydids using pure-tones above 10 kHz (55).

### The Evolution of Hearing and Singing.

**Fig. 5.**
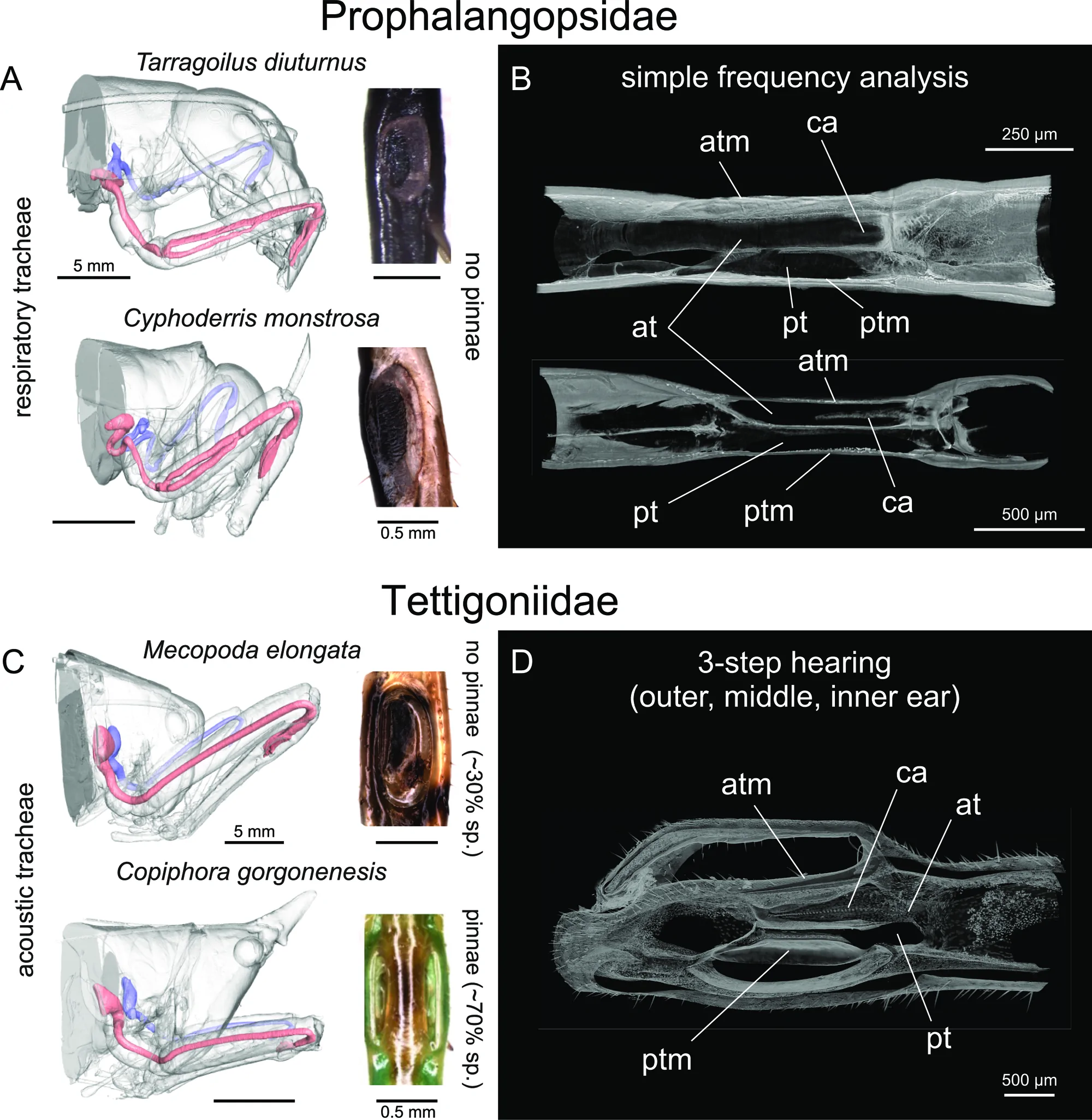
The hearing system of extant grigs (Prophalangopsidae) and katydids (Tettigoniidae). (*A*) The bifurcated trachea in the forelegs of *T. diuturnus* and *C. monstrosa*, unspecialized for the transmission and capture of sound. (*B*) The simplified inner ear of the species in A, showing a plesiomorphic version of a *crista acustica*, performing frequency analysis aided by the tympana. (*C*) The acoustic trachea, or ear canal, in extant katydids, displaying the common forms of tympana: naked or exposed (*Mammillaria elongata*), and covered by auditory pinnae (*Copiphora gorgonensis*). (*D*) longitudinal section of a *C. gorgonensis* ear, showing the elaborated inner ear (or insect “cochlea”), specialized for frequency analysis using frequency mapping and traveling waves. atm: anterior tympanic membrane; at: anterior tracheal branch of the acoustic trachea; ca: *crista acustica*; ptm: posterior tympanic membrane; pt: posterior tracheal branch. Scale for ears in *A* and *C*: 0.5 mm; scale for tracheae in *A* and *C*: 5 mm.

Driven by natural and sexual selection, insect ears evolved mainly for intraspecific communication and predator detection (56). In classical studies of insect hearing, it has been a chicken-and-egg question of what evolved first: the ability to sing or the ability to hear? (44). Modern katydids (Tettigoniidae) are unique among arthropods for having evolved the three canonical steps of hearing as used by mammals. Their hearing system is endowed with outer, middle, and inner ear components (57). Frequency analysis is achieved in the inner ear by means of tonotopy (frequency mapping) and traveling waves (57, 58). The inner ear is made of a sensory organ, the *crista acustica* (CA) which, in living species, contains between 15 and 116 auditory sensilla (59) (Fig. 5 *C* and *D*), and in a large number of species is enclosed in a fluid-filled cavity known as the auditory vesicle. In the extant Prophalangopsidae the inner ear structure is much simpler (Fig. 5 *A* and *B*).

**Fig. 6.**
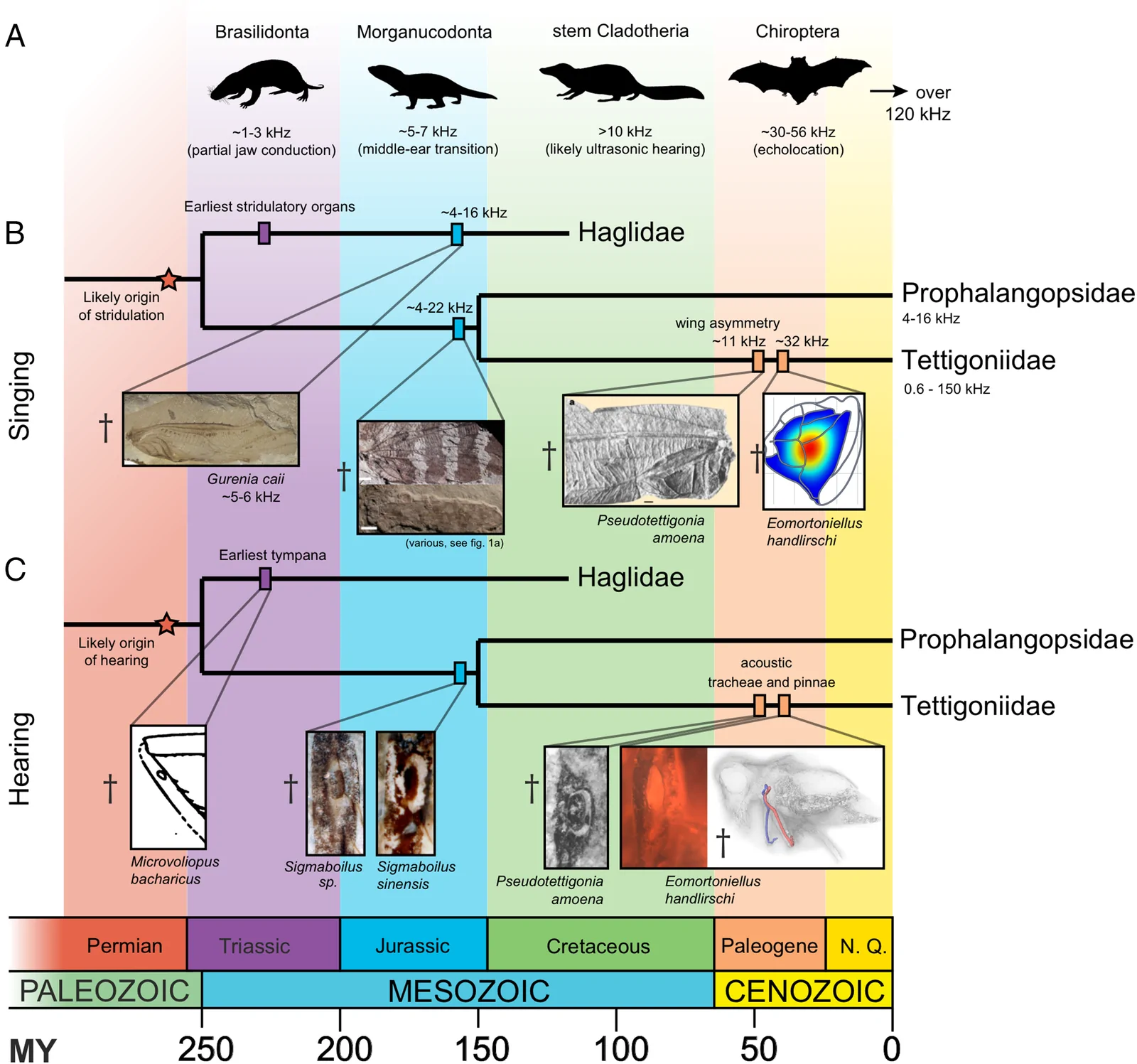
Proposed scenario for the evolution of singing and hearing in grigs (Prophalangopsidae) and katydids (Tettigoniidae). (*A*) The evolution of auditory sensitivity in mammals and non-mammalian ancestors. (*B*) Evolutionary map of sound production by stridulation in Tettigoniidea. (*C*) Evolutionary scenario for hearing in Tettigoniidea. (*) Hearing organs adapted from: *M. bacharicus*: Gorochov (66). *A. hani*: Gu et al. (67). *Sigmaboilus. *spp*.*: Xu et al. (3). *P. amoena*: Rust et al. (26). *E. handlirschi*: Woodrow et al. (33) (†). Wings adapted from: *P. amoena*: Rust et al. (26). *E. handlirschi*: Woodrow et al. (33). N. Q. = Neogene and Quaternary.

The ability to produce sound by wing stridulation could have appeared before hearing as a defensive tool to deter terrestrial predators like reptiles (60) and insectivorous mammals, including gliding species (61). Many living katydids produce disturbance sounds to deter predators (62). An interesting example where disturbance sounds are produced, but one’s own ability to hear such signals is lacking, comes from the living Prophalangopsidae, *Cyphoderris monstrosa*, which produces broadband ultrasound (peaking at about 60 kHz) by tergo-tergal stridulation. Yet, male and female *Cyphoderris* spp., cannot hear these ultrasounds (63), and therefore this defensive behavior is hypothesized to deter insectivorous mammalian predators such as shrews (64). This exemplifies the possibility that sound production can evolve without ears, as well as the widespread presence of stridulatory organs in insects without ears (15). A primitive stridulatory organ in the forewing of early Ensifera could have been established by the Upper Permian in the family Oedischiidae (65), however the clearest evidence of a fully formed stridulum comes from the middle Triassic. Tympanal organs are known from this period as well (66) (Fig. 6). So, if a form of primitive stridulation emerged in the upper Permian, it is likely that sound production predated the ability to hear using tympanic membranes. However other studies have argued that based on the presence of tympanal organs in both major clades of the Ensifera (Grylloidea and Tettigonioidea), singing and hearing may have coevolved in this group (7). The evolution of a single stridulatory file, with regularly spaced teeth is a clear departure or improvement from the relic condition of stridulation, with multiple randomly distributed teeth or pegs (*SI Appendix*, Fig. S7) (25). The adoption of a single stridulatory file with regularly spaced teeth, along the CuPb vein (31), was an important step for the evolution of more elaborate calls with narrower frequency bands. This file structure must then be accompanied by a corresponding single elastic plectrum (as opposed to multiple files and hard scraping regions (25).

Certainly, singing in the presence of eavesdropping predators was dangerous, and one way to call safely was to narrow the bandwidth of the call. This is because pure tones are challenging to localize with the mammalian ear. In living mammals, the cochlea spectral differences that provide directional hearing cues are limited by a quality factor (*Q*_-3_) of about 9 to 13. In many living katydids and all grigs, pure-tone songs exhibit *Q* > 10 (including the species shown here), implying that natural selection for pure-tone calls happened earlier in the insect-predator race. The cochlea of Jurassic mammals might have had a lower demand for pure-tone directional hearing, and thus lower *Q* resolution, making narrow-band calling an efficient strategy for early katydids to avoid eavesdropping predators. For eavesdropping strategies to remain functional, mammalian hearing resolution needed to improve, which gave rise to the earliest known acoustic arms-race. With short, uncoiled cochleae, these mammals had a limited frequency hearing range (38, 68) (Fig. 6*A*). Therefore, successfully selected eavesdroppers might have been those who coped with at least one of the two new strategies of the evolving prey. The hearing system in the ancient Ensifera should have gradually adapted to the musical but high-frequency tendency of conspecific calls (e.g., *S. peregrinus*), while continuing to function in detecting low-frequency sounds for environmental cues and predator detection. These combined pressures lead to the evolution of a more sophisticated frequency analysis organ — the insect cochlea (57).

The inner ear of extant Prophalangopsidae (e.g., *Cyphoderris* spp. and *Tarragoilus diuturnus)*, considered “living fossils”, are vital to understand this process. The CA of *Cyphoderris* and *Tarragoilus* is very simple when compared to that of Tettigoniidae (Fig. 5). It has been recently shown that *Cyphoderris monstrosa* achieves frequency mapping via traveling waves on the tympanic membrane (69). In other words, the mechanoreceptors of a precursor CA in living Prophalangopsidae are neither completely isolated from the tympanic membrane nor enclosed in a fluid-filled vesicle, as in Tettigoniidae (57).

Another fundamental component of the hearing system in living katydids is the acoustic trachea (or ear canal), which serves to augment gain and funnel sound to the tympanal organ and to enhance directional hearing sensitivity via a pressure-difference receiver system (70). Fig. 5*A* shows the relic conditions of the acoustic trachea (bifurcated), which is not functional for sound transmission in the living Prophalangopsidae (*Cyphoderris* and *Tarragoilus*) but has been adapted as a sound guide in most living katydids (Fig. 5*C*). A bifurcated auditory trachea seems to be the plesiomorphic condition of a functional acoustic trachea (*SI Appendix*, Fig. S7). Although the acoustic trachea is not preserved in fossilized Jurassic specimens, it is very likely that it was not specialized for sound transmission, as observed and measured in *Cyphoderris* and *Tarragoilus* (Fig. 5*A*), at least in species singing below 12 kHz. The most common form of acoustic trachea currently known in living katydid species is the exponential horn type, which serves to passively amplify sound waves and delay their propagation, favoring a more accurate sound source localization in the form of a pressure difference receiver (70). The earliest record of this exponential-horn outer ear has recently been shown to have evolved in the Eocene (33) (Fig. 6), showing acoustic sensitivity between ~10 kHz and 89 kHz, which covers the frequency range of the male calling song and the echolocation calls of early bats.

These facts leave us with two hypotheses: 1) Jurassic ensiferans were able to detect a broad range of frequencies from low audio to moderate ultrasound, using the external surface of the tympanic membrane, without the passive amplification function of the acoustic trachea, or 2) some species had started to evolve an ear canal with specialized geometries for broadband sound amplification, such as an exponential horn. The first hypothesis implies that species would have required a specialized CA able to analyze a broad range of frequencies, which is not the case in living but relic Prophalangopsidae (Fig. 5*B*), detecting frequencies below 13 kHz. The second hypothesis implies that species enhanced their directional hearing in the ultrasonic range, and improved predator detection of insectivorous mammals. The incorporation of high frequencies requires a more sophisticated sound-capturing system (e.g., ear canals, pinnae, and CA for frequency analysis, as shown in modern katydids, Fig. 5) than in extant grigs (Prophalangopsidae).

For now, we can only confirm that Jurassic ensiferans were communicating with a broad range of frequencies from low audio to moderate ultrasound. We show that ultrasonic communication was likely adopted by katydid ancestors during the Middle Jurassic, some 165 ma, the oldest record known for ultrasound communication in animals. In addition, these ancient ensiferans had already started to diversify in their acoustic signaling strategies, through changes in body size and stridulatory file morphology, producing pure-tone and high-pitch calls. While the observed diversity of extant katydid acoustic signaling strategies and the predominant use of pure-tone ultrasound are thought to have been driven by predator eavesdropping (35, 71), we here reject the hypothesis that bats were the sole driver of ultrasound evolution in katydids. Instead, it is likely that early mammals and nonmammalian ancestors were also listening in to the songs of ensiferans, driving early diversification of acoustic signaling strategies. The evolution of ultrasound could have also been driven by acoustic niche partitioning due to interspecific competition. This, combined with evidence for ultrasound in Cretaceous moths (72), implies that bats emerged – nearly 100 Mya after singing ensiferans – into a soundscape already busy with ultrasounds.

## Materials and Methods

### Wing Geometry and Material Properties.

Forewing geometries were reconstructed by tracing wing-venation illustrations over scale-calibrated fossil photographs in Adobe Illustrator (v2023). Wing damping in fossils was estimated using experimental data from living species. Using LDV, we derived *Q* from wing oscillations or from free decay after a single tooth strike. Wing resonances also allowed us to estimate Damping coefficient (ζ). Both parameters were implemented in the numerical models to recover vibrations in fossilized wings. Micro-CT scanning and 3D segmentation follow established protocols (*SI Appendix*, *Methods*).

### Sound and Wing Motion Recordings.

Acoustic and wing motion recording are described in Woodrow et al. (2). Protocols for high-speed video are described in *SI Appendix*, *Methods*.

### Finite Element Analysis (FEA).

The wing acoustic properties were simulated using the finite element simulation toolbox COMSOL Multiphysics, v. 6.1 (73). The mathematical models used the reconstructed wing geometry described in the section *Wing Geometry* above as the solution domain. The actively vibrating wing areas (wing membrane) were modeled using a linear shell formulation of COMSOL Multiphysics (73), and were assumed to be isotropic and homogeneous. Wing membrane areas were selected based on the coherent active vibrating areas in the extant Prophalangopsidae Including *Prophalangopsis obscura* (17), *Cyphoderris monstrosa* (27), and the closest living relatives to the Ensifera of the Jurassic studied here, for example, *Tarragoilus diuturnus* (Fig. 2). For further details of the FEA transition between living and fossilized wings see *SI Appendix*, *Methods*.

### Phylogenomic Analysis.

#### DNA Extraction.

To obtain high–molecular–weight genomic DNA (≥1 µg per sample) for target enrichment and Illumina sequencing, we extracted DNA using the Gentra Puregene Tissue Kit (Qiagen) according to the manufacturer’s protocol. DNA yield and purity were first assessed with a DeNovix spectrophotometer, then with the Qubit 2.0 fluorometer.

#### Target enrichment library preparation and sequencing.

We generated hybrid capture (target-enrichment) sequence data following the general methodology of Shin et al. (42). These are single-copynuclear protein codinggenes, originally identified from orthopteran transcriptomes. Briefly, genomic DNA was converted into Illumina-compatible libraries using an NEBNext® Ultra™ II FS DNA Library Prep Kit for Illumina, after which libraries were enriched using a custom OR-TE probe set (42) developed for Orthoptera and synthesized for use with myBaits® Custom Hybridization Capture Kits for targeted DNA sequencing from Daicel Arbor Biosciences (Arbor Design ID D10583GRHP2). Enriched libraries were pooled and sequenced on an Illumina NovaSeq 6000 platform to generate paired-end reads.

#### Data processing, filtering, and phylogenomic analysis.

We followed methods described in Shin et al. (42) for raw read processing, contig assembly, orthology assignment, data cleaning, and alignment to create an input data for our phylogenomic analysis. We filtered alignments and removed initial transcriptomic biases using custom python scripts. To assess gene-level topological variation and identify problematic taxa, we inferred a gene tree for each filtered locus alignment in a maximum likelihood framework. We examined patterns of concordance and conflict among loci and identified sequences exhibiting anomalous placement likely driven by missing data, alignment artifacts, or contamination using an established pipeline. We concatenated the final 1,077 locus alignments into a supermatrix and generated a best-fit partitioning scheme for our dataset. We inferred a maximum likelihood phylogeny and with nodal support valued assessed with 1,000 ultrafast bootstrap replicates. See *SI Appendix*, *Method*s for fine details.

### Divergence-Time Estimation Using Fossil Calibrations.

We estimated divergence times using MCMCtree (PAML v4.10.5) (74) within the MDGUI package of PhyloSuite v2 (75) with a curated set of fossil calibrations selected on the basis of previous, well-supported Orthoptera phylogenies (7) (*SI Appendix*, Table S6). These were justified following the logic laid out in Song et al. (7) To accommodate uncertainty associated with a sparse and uneven fossil record, we implemented soft time constraints using broad skew-t prior distributions parameterized in MCMCtreeR (76). Dating analyses used a GTR substitution model on a concatenated supermatrix comprising 214 loci selected to be high-coverage and approximately evenly distributed across evolutionary rates. We used the Hessian approximation to estimate the gradient/Hessian and ran four independent MCMC chains, each for 4,000,000 iterations (with burn-in removed prior to combining). Convergence and mixing were assessed in Tracer v1.7.2 (77) by evaluating trace stationarity and effective sample sizes (ESS); all chains achieved ESS > 200 for all parameters. Because acoustic data were available for only a subset of genomically sampled taxa, we first time-calibrated the phylogeny using the full taxon set to maximize dating robustness, and then pruned the tree to the acoustics-enabled subset for downstream phylogenetically informed analyses (*SI Appendix*, Fig. S10).

### Estimating Fossil Syllable-Rate Using Gaussian Process Regression.

Syllable rates of fossil orthopteran insects were estimated using a probabilistic Gaussian Process Regression (GPR) framework trained on extant species (78) (*SI Appendix*, Fig. S9 *B* and *C*). Four predictors were used: stridulatory file length, total tooth number, dominant frequency, and syllable duration. The target variable (syllable rate) was calculated at a standardized ambient temperature of 20 °C using published temperature–rate equations. Because syllable rate values are strictly positive and span an order of magnitude, the response variable was log-transformed prior to modeling.

A composite kernel combining a Matérn covariance function, a linear DotProduct component, and a WhiteKernel noise term was implemented to capture nonlinear relationships while preserving extrapolative capacity. Kernel hyperparameters were optimized using Bayesian optimization implemented via the Optuna framework (79) with cross-validation. Model performance was evaluated using conventional regression evaluation metrics.

The validated model was applied to fossil taxa using reconstructed morphological and acoustic inputs. Posterior predictions were exponentiated to return values in physical units and accompanied by uncertainty estimates derived from the GPR posterior distribution. Predicted syllable rates were compared against biomechanical maxima derived from syllable duration to ensure biological plausibility. See *SI Appendix*, *Methods* for more details.

ChatGPT (GPT-4o, OpenAI) was used during code development to assist in establishing the initial architecture of the GPR framework and the Optuna-based hyperparameter optimization script. All AI-assisted code and resulting model performance were manually reviewed, debugged, and biologically validated by the authors against known insect syllable rate benchmarks and physical constraints. AI software was not used in the writing, drafting, or editing of the manuscript text.

## Supplementary Material

Appendix 01 (PDF)

Movie S1. High-speed video recording of a stridulating male Gryllus bimaculatus, captured at 10,000 frames per second. This footage offers a striking window into the mechanics of stridulation, revealing how the cricket shapes and controls its carrier frequency in real time. It’s an invaluable visual tool for understanding both the precision and the complexity of acoustic signaling in this species.

Movie S2. Wing-vibration animation of a male field cricket’s forewing, generated through a numerical model using a simplified two-dimensional wing geometry. This models serve as a proof-of-concept, allowing us to compare its predictions with the real vibrational behavior of the same insect’s wing, measured through Laser Doppler Vibrometry. The numerical model requires investigating the wing’s material properties, which determines how faithfully the model can capture the true dynamics of the living structure.

Movie S3. Wing-vibration animation of a fossilised forewing from the Jurassic ensiferan *Sigmaboilus peregrinus*, generated through a numerical model using the two-dimensional wing geometry approach described in Movie 2. The simulation uses material properties measured from living species, allowing us to explore how this ancient wing might have behaved in life and to draw tentative comparisons between fossil morphology and the vibrational mechanics of modern relatives.

Movie S4. Reconstructed katydid ancestor calls from the Jurassic, matching a each species wing, used in the reconstruction, with the respective call obtained.

Movie S5. Wing resonance of a male *Tarragoilus diuturnus*, one of the few relic living ensiferans, showing the main wing cells responsible for producing this species’ characteristic ~5 kHz call. The wings were stimulated sympathetically using broadband sound, and their vibrational response was captured with a Micro-Scanning Laser Doppler Vibrometry. The result highlights the remarkable precision and acoustic specialization preserved in this ancient lineage.

Movie S6. Measure of wing displacement amplitude of the right forewing of the relic living Ensifera, *Tarragoilus diuturnus*. The wings have been stimulated sympathetically with broadband sound, and recording using a Micro-Scanning Lasser Doppler Vibrometry, as explained for Movie 5. Profile dissecting lines show the displacement of the main wing area involved in sound radiation.

Audio S1. Reconstructed katydid ancestor calls from the Jurassic.

Code S01 (TXT)

Code S02 (TXT)

Code S02A (TXT)

Code S02B (TXT)

Code S02C (TXT)

## Data Availability

Wing geometries used for numerical modeling, and COMSOL models have been deposited in Zenodo: https://doi.org/10.5281/zenodo.19882665 (80). All other data are included in the manuscript and/or supporting information.
