# Supplementary material for: Reconstruction of an extinct soundscape reveals ultrasonic communication in the Jurassic
Source: Proc Natl Acad Sci U S A. 2026 Aug 25;123(36):e2615107123. doi: 10.1073/pnas.2615107123 (PMC13552889; doi:10.1073/pnas.2615107123)
Supplement: Supplementary file 1 [file pnas.2615107123.sapp.pdf]

PNAS

Supporting Information for:

Reconstruction of an extinct soundscape reveals ultrasonic communication in the Jurassic

Authors: Jun-Jie Gu[1,2*†], Fernando Montealegre-Z[3*†], Thorin Jonsson[†4], Charlie Woodrow[3], Emine Celiker[5], Md Niamul Islam[3], Jackson B. Linde[6], Fabio Sarria-S[3], Fuming Shi[7], Hojun Song[6], Daniel Robert[8] &, Dong Ren[2*]

*Correspondence to: FM-Z: fmontealegrez@lincoln.ac.uk, JJG: orthoptera_gu@aliyun.com, or DR: rendong@mail.cnu.edu.cn

This PDF file includes:

Supporting text
Figures S1 to S10
Tables S1 to S7
Legends for Movies S1 to S6
Legends for software S1 to S2

SI References

Other supporting materials for this manuscript include the following:

Audio S1
Movies S1 to S6
Software S1 to S2

Supporting Information Text

1. Supplemental Methods

A. Wing geometry and material properties

A.1. Wing venation maps. To reconstruct the geometries of the forewings, illustrations of the wing venation were traced over scale-calibrated photographs of the fossil specimens in Adobe Illustrator v2023 (Adobe Inc., San Jose, California, United States). The lines were made to be vector based, and the forewing built as a .DXF file and imported into the xy plane of the numerical simulation software.

A.2. Wing Damping calculations. Wing damping in fossils was estimated using experimental data from living species. For small damping $\zeta<1$, which means the decay is oscillatory. If $\zeta=1$, the damping is critical and the displacement tends to zero in the shortest possible time. ζ varies from undamped ($\zeta=0$), underdamped ($\zeta<1$), through critically damped ($\zeta=1$) to overdamped ($\zeta>1$). ζ is a function of the Logarithmic decrement of the oscillation, therefore it is also a function of *Q*. We calculated both ways in several species (*Gryllus bimaculatus*, *Cyphoderris mostrosa*, *Tarragoilus diuturnius*, *Docidocercus sagittatus*, and *Panacanthus pallicornis*) with various calling frequencies, using LDV, we calculated *Q* from the wing oscillation, or measuring the free decay or the oscillation produced by a single tooth strike (click), and found no differences (Table S3). Thus, both ways are good indicators of ζ.

From the calculated numbers below, for left and right wings, ζ ranges from 0.03 to around 0.08, suggesting these Ensifera wings tend to be underdamped ($\zeta<1$). We suggest fossil artificial oscillations decay around the values reported in Table S3.

B. Phylogenomic analysis

B.1 Data processing, filtering, and phylogenomic analysis. Adapter contamination and low-quality bases were removed using Trimmomatic (1), and trimmed reads were assembled de novo using SOAPdenovo2 (2). To assign assembled sequences to orthologous loci with high specificity, we used Orthograph with an Orthoptera-focused ortholog database. Orthograph (3) was used to screen assemblies and identify putative

orthologs, thereby minimizing inclusion of paralogs and non-target contaminants while standardizing locus identities across samples. Thousands of recovered loci are generated at this step.

B.2. Ortholog alignment construction and orthology quality control. Orthologous sequences were subsequently processed through the 1KITE cleaning pipeline to further curate orthology and standardize locus sets (4). This step included additional orthology checking and cleaning procedures, followed by multiple sequence alignment of each orthologous locus using MAFFT (v7.130b) (5). The resulting per-locus alignments formed the initial matrix for downstream filtering and phylogenetic inference.

B.3. Site-level filtering and sequence-level cleanup. We filtered alignments a custom python script (01_filter_gap_alignments.py) allowing up to 90% gaps per column of the alignment, and up 35% missing data (within a solid empirical threshold)6 per row with a minimum of 100bp and at least 4 taxa present - this was done as utilizing small alignments and limited filtering allows for true signal to be retained (6). After filtering, to deal with initial transcriptomic biases (these data consist of thousands more genes than typical hybrid capture, and can thus overwhelm the lesser amount of data and transcriptomic data can be drawn together artificially) we used our custom transcript_only_filter.py to remove alignments consisting of only transcriptomic sequences.

B.4. Gene tree inference and rogue taxon mitigation. To assess gene-level topological variation and identify problematic taxa, we inferred a maximum likelihood gene tree for each filtered locus alignment in RAxML-NG v1.2.0 (7). These were run with the GTR+G model and 100 bootstrap trees. These per-locus trees were used to examine patterns of concordance and conflict among loci and to identify sequences exhibiting anomalous placement likely driven by missing data, alignment artifacts, or contamination. We then applied TreeShrink v1.3.9 (8) to the gene tree set to detect and mitigate the impact of rogue taxa (i.e., taxa producing unusually long branches or unstable placements across loci). Taxa flagged as likely to distort inference were removed or down-weighted according to the TreeShrink workflow (8), thereby improving robustness of downstream concatenated and time-calibrated analyses. After TreeShrink, we took updated alignments (adjusted by the program) and re-estimated gene trees with rogue taxa removed.

B.5. Monophyly screening to prevent technology-driven clustering. Because our dataset combines hybrid capture and transcriptome-derived sequences, we implemented an additional safeguard to ensure that sequences were not clustering artificially by data type rather than evolutionary history. We used a custom monophyly filter (final_monophyly_checker.py) to test whether transcriptome-derived sequences formed spurious monophyletic groups across loci or in the concatenated topology. Loci or taxa showing evidence of technology-associated, artificial monophyletic grouping by data type were flagged and removed prior to final inference, similarly to Frandsen et al. (9).

B.6. Concatenated phylogenomic inference and partition optimization. We concatenated the final 1,077 locus alignments into a supermatrix using AMAS.py script (10) and generated an initial partitioning scheme with one partition per orthologous locus. We inferred the maximum likelihood phylogeny of the 625,593 bp supermatrix in IQ-TREE v3.0.1 (11) using comprehensive model and partition optimization. Specifically, we used ModelFinder (12) with partition merging enabled (MFP + MERGE) and the greedy algorithm (rcluster) with parameter 10, allowing the data to determine an optimised, reduced partition scheme (13). Node support was assessed with 1,000 ultrafast bootstrap replicates (14) with the "-bnni" flag added to reduce the impact of known ultrafast bootstrap inflation toward values of 100 and 1,000 SH-aLRT replicates, providing complementary measures of branch reliability.

B. Sound recordings and wing mechanics

B.1. Acoustic and wing motion recordings. Stridulatory wing movements and the associated sound production were recorded from *Cyphoderris monstrosa* (3 males), *Tarragoilus diuturnus* (2 males), *Gryllus bimaculatus* (9 males), and, and *Panacanthus pallicornis* (8 males) using protocols well established by the authors. Acoustic signals were captured using a calibrated 1/8″ microphone (Brüel & Kjær Type 4138 connected to a preamplifier Type 2633) and amplified with a B&K1708 conditioning amplifier (Brüel & Kjaer, Nærum, Denmark). Wing movements were monitored using an opto-electronic device (15, 16). To facilitate recordings from dorsal or posterior views, a piece of reflective tape (Scotchlite 7610 and 8850, 3M) was affixed to the left tegmen (15). Sound and wing movement signals were recorded on separate channels using PSV acquisition software (Polytec GmbH, Waldbronn, Germany), which employed a National Instruments (NI)

acquisition board. A high-pass filter was set at 2 kHz for the sound recordings, a low-pass filter with a cut-off at 5 kHz for the wing motion, and a sampling frequency of 512 kHz. Experimental conditions were maintained at a temperature of 24-26 °C and a relative humidity of 35–40%.

Wing motion in stridulating insects were also obtained using a Photron FastCam Mini at 5000-15000 frames per second with a shutter speed of 1/6000. Persistent stridulation in male crickets (*Gryllus bimaculatus*) was elicited using pharmacological stimulation following the protocols of Wenzel and Hedwig (17), and Jonsson et al. (18).

B.2. Laser Doppler Vibrometry. Wing resonance was measured in the same species above [*Cyphoderris monstrosa* (3 males), *Tarragoilus diuturnus* (8 males), *Gryllus bimaculatus* (47 males), and, and *Panacanthus pallicornis* (8 males)], using micro-scanning laser Doppler vibrometry (LDV; PSV-500, Polytec GmbH, Waldbronn, Germany). In these experiments, the insect was mounted on a horizontal brass platform following the methodology outlined by Montealegre-Z et al. (16). The dorsal region of the wings was stimulated using synthetic sound and scanned with the LDV across a grid of approximately 1,000 points, utilising a sample rate of 128 kHz and averaging three measurements per point. The acoustic stimulation comprised broadband periodic chirps within the frequency range of 1.5-50 kHz. The sound pressure of the stimulus was calibrated to ensure a consistent sound pressure level (SPL) of 60 ± 1.5 dB (re. 20) throughout the experimental range. Acoustic signals were generated by the internal data acquisition board of the PSV (PCI-4451; National Instruments, Austin, TX, USA), amplified (A-400, Pioneer, Kawasaki, Japan), and transmitted to a loudspeaker (Ultrasonic Dynamic Speaker Vifa, Avisoft Bioacoustics, Glienicke, Germany) located 30 cm from the specimen. The reference signal was recorded with a 1/8″ condenser microphone placed horizontally near the wings (Brüel & Kjaer, 4138-A-015, and preamplifier model 2670, Brüel & Kjaer, Nærum, Denmark).

C. Micro structure of the hearing system in living forms

C. 1. Micro-computed Tomography. Specimens were scanned using a SkyScan 1172 μ-CT scanner (12.9 μm voxel size, 55 kV source voltage, 200 μA source current, 200 ms exposure, 0.2◦ rotation step; Bruker Corporation, Billerica, MA, USA). μ-CT projection images were reconstructed to produce orthogonal slices with NRecon (v.1.6.9.18, Bruker

Corporation, Billerica, MA, USA). For 3D segmentation of tracheae, the orthogonal slice data were imported into Amira-Aviso 6.7 (Thermo Fisher Scientific, Waltham, Massachusetts, USA), and the lumen of the tracheae was selected using the magic wand tool every 5 slices throughout the whole dataset, followed by interpolation to connect slices. This selection was then used to generate a 3D surface and 2D image. The 3D image of the inner ear of the katydid (*Mimetica* sp.) is modified from Celiker et al (19).

D. Numerical modelling

D.1. Finite Element Analysis (FEA). Wing membrane areas were selected based on the coherent active vibrating areas in the extant Prophalangopsidae Including *Prophalangopsis obscura* Woodrow et al. (20), *Cyphoderris monstrosa* (21), and the closest living relatives to the Ensifera of the Jurassic studied here, for example, *Tarragoilus diuturnus* (Fig. 2 main text). This region of vibration is restricted to specific cells of the forewings, and thus we used the corresponding wing veins surrounding the outer-most margin of these cells to mark the edge of the vibrating areas, as learned from LDV experiments in *T. diuturnus* (Fig. 2 main text) and *C. monstrosa* (21). In *P. obscura* and *T. diuturnus*, on the other hand, this vibration extends from the CuPb vein to the margins of the CuPaβ and the CuPaα veins (Fig S5, Movie S5) Woodrow et al. (20). In *C. monstrosa*, this region extends from the CuPab vein to the margins of the CuPa and the CuPaa2 veins, and corresponds to the mirror, pre-mirror, and neck areas, with no contribution from the harps (21). As the forewings of extant Prophalangopsoidae *P. obscura*, *Aboilomimus sichuanensis* and *T. diuturnus* are similar in geometry to those of the fossils studied here (Fig. S5) and are estimated to have similar biophysical properties Woodrow et al. (20), we used the wings of these species to determine the scope of the vibrating area. The geometry of the vibrating area is relevant here, and, contrary to early research (22, 23), this area is not limited to a vein enclosure, such as the mirror of katydid wings, or the harp in crickets. The vibrating area in the Ensifera wings extends to other surrounding areas where vibration coherence is met (18, 21, 24).

The forewings of male Ensifera are substantially thicker than the hind wings; the values on thickness and elasticity are known from a few published papers on cricket (25) and locust (26) wings, and cannot be known from compression fossils. Thus, to estimate wing thickness, we used empirical measurements of Young's modulus (E) from the thickest part

of the locust and cricket hind wings from references above (~9 GPa) and conducted a manual parameter sweep of membrane thickness until the resulting first natural frequency matched the frequency predicted by regression models of the stridulatory file length (see Eigenfrequency studies below). It should be noted, therefore, that variation in E is not considered in these reconstructions. This resulted in variation in wing membrane thickness within the range of 5.5-18 μm (Table S4). As a proof of concept, the values were validated and used to recover wing vibration from extant species (*Tarragoilus diuturnus, Panacanthus pallicornis, Gryllus bimaculatus*), where the physical reality of wing vibration was previously known from Laser Doppler vibrometry experiments (see Fig. 2, main text).

The veins on the wings were represented by a linear beam formulation and were also assumed to be isotropic and homogeneous. The veins were assigned the same elastic modulus of 9 GPa, and a thickness multiplier of 3 μm. This allowed us to consider that the veins are thicker than the wing membrane, but without having to specify the thickness of every vein individually. Based on fossil images and extant Prophalangopsidae, specialised veins do not appear to be present. Finally, the wing membrane and the veins were coupled using the Shell-Beam Connection feature of COMSOL Multiphysics, v. 6.1 (27). We highlight that further information on the material properties of extant taxa will be crucial in refining these modelling-based approaches in the future. Our validation with extant taxa supports our current methodology. Table S5 lists the material properties incorporated into the mathematical models for the wings.

The acoustic properties of the fossilized wings were investigated using an eigenfrequency study and by considering the problem in both the frequency and time domains. For all three problems, the solution to the variational form of the considered shell-beam formulation was obtained using the finite element method.

The element size of the formed finite element mesh was determined based on a mesh sensitivity analysis carried out separately for each species. For this analysis, the mesh size was systematically refined to check for the mesh dependence of the solution. Based on this analysis, the mesh was given a maximum element size between 4-2 mm and a minimum element size of 0.5-0.2 mm. This mesh size also ensured that there were at least six finite elements per wavelength when the frequency is 1-30 kHz. While the speed of sound in the

wing is not known, it is expected that in such a membranous structure as the fossil wings, it is around 1500 m/s. Fig. S9a demonstrates the global mesh refinement carried out for the frequency domain solution of *Archaboilus polyneurus*. The maximum element size was reduced from 5 mm to 1.5 mm. As there was no significant difference in the solution after the element size was smaller than 2.5 mm, the maximum element size for this species was taken as 2.5 mm. A similar procedure was carried out for all the species.

D.2 Eigenfrequency Studies. The natural frequency and harmonics of the wings were calculated using COMSOL Multiphysics, v. 6.1. The discrete solution of the eigenfrequency study was obtained with the finite element method, where the considered system of finite element equations was solved using the eigenfrequency solver ARPACK built in COMSOL Multiphysics, v. 6.1.

D.3. Frequency Domain Simulations. The system was excited by applying a fixed load to a vein within the actively vibrating wing area. The frequency range of the load was 1-30 kHz with a resolution of 0.5 kHz. The magnitude of the applied load was taken as 2.5e-5 N for all species. The magnitude was chosen based on the force applied during analogous experiments. Because the models are linear, however, the magnitude of the applied force is not of major significance for this study.

A fixed constraint was placed around the active wing area to ensure a stable solution. The problem was discretised using the finite element method as described above. Finally, the system of finite element equations was solved using the stationary solver MUMPS.

The solutions were obtained by recording the maximum displacement magnitude within the active membrane region at each frequency. The solution was then normalized with respect to the maximum displacement magnitude for each species.

D.4. Time Dependent Studies. To construct the fundamental syllable of the fossilised wings, numerical models were developed to simulate the wing vibrational response to an applied force on one of the veins within the active region of the wing. The model was set up using the coupled shell and beam formulations of COMSOL Multiphysics in the time domain. Using the edge load condition, a vein on the wing was excited with a force of magnitude 2.5e-5 N for all species. At each time step, the solution was obtained by the finite element method, as described in Veitch et al.(19). The time step was taken as

$\Delta t=1/(128\ \text{kHz})=7.8125\times10^{-6}$ s,

and the simulation had a duration of 0.004 s. For the time discretization, the generalised alpha method was employed Veitch et al.(28).

E. Prediction of syllable rates in fossilised insects

E.1 Gaussian process regression. This study employed a machine learning approach to estimate the syllable rates of calling song of extinct orthopteran insects based on preserved the morphological and the simulated acoustic traits. Gaussian Process Regression (GPR) was selected as the core modelling technique due to its non-parametric, probabilistic framework, as it does not impose a fixed functional form on the data. GPR is well-suited to small-to-medium-sized biological datasets, making it appropriate for the limited number of extant species available for training (29). Additionally, it enables the generation of confidence intervals alongside point predictions, allowing quantification of uncertainty, an important requirement when extrapolating to fossil taxa. The ability of the model to capture non-linear relationships while extrapolating to regions beyond the training data make GPR particularly effective for reconstructing extinct calling song.

E.2. Training Dataset and Features**.** The training dataset was compiled on four input features based on the morphological and the acoustic traits of extant and extinct species:

1. The stridulatory file length (mm)
2. Total number of teeth on the stridulatory file
3. The dominant frequency (kHz) of the species' acoustic signa
4. The syllable duration (ms) in the call.

The target variable was the syllable rate, calculated from the equation of 'syllable/wingstroke/phanotome rate changes with temperature' from existing literatures (Table S7) (30–38). An ambient temperature of 20 °C was selected for the syllable-rate calculations as it sits near the centre of Jurassic climate estimates from multiple lines of evidence. Global multi-proxy compilations place mean surface values between ~19.6 °C (Early-Jurassic cool interval) and ~24.6 °C (Toarcian warm interval) (39); high-latitude TEX-86 records from the Falkland Plateau yield consistently warm sea-surface temperatures of 26–30 °C during the Middle-to-Late Jurassic (40); and a mid-latitude continental reconstruction for the Callovian Yanliao Biota indicates mean annual air

temperatures of 15–20 °C (41). A value of 20 °C therefore provides a representative midpoint for the range of Jurassic ambient conditions. Some species exhibited two distinct syllable rate–temperature equations, from which the higher syllable rate was selected for modelling to ensure a consistent and conservative estimate of the species' maximum acoustic performance. This approach establishes an upper bound for communication rate based on available data and avoids underestimating the insect's potential signalling capacity. Species with complex phanotomes (calling song consisting of multiple syllable and different syllable rate equation for different syllables) were ignored to reduce complexity of the model for prediction.

E.3. Kernel Construction. The GPR model's behaviour is governed by its kernel (covariance function), which defines similarity between data points. A composite kernel was constructed:

$$k(x, x') = c_1 \cdot Matern_v(\ell) + c_2 \cdot DotProduct + WhiteKernel$$

- The Matérn kernel $Matern_v(\ell)$ was used for modelling spatial smoothness, where ℓ is the characteristic length scale and $\nu \in \{0.5, 1.5, 2.5\}$ controls smoothness (from rough to smooth functions). The scaling constant c_1 modulates the variance of the function prior, essentially controlling how much influence the Matérn component has on the output. Larger values of c_1 allow the model to capture higher variability driven by smooth patterns in the input space. This kernel was chosen to capture local non-linearities while providing a tuneable smoothness prior, which has been effective in ecological and evolutionary models.

- The DotProduct kernel, scaled by c_2, captures linear relationships and supports extrapolation, which is critical when making predictions for fossil data lying outside the range of the extant species. The scaling constant c_2 defines how much weight this linear trend contributes to the overall model.

- A WhiteKernel captures small observational noise, allowing for non-zero variance even when inputs are identical and avoid overfitting.

Together, c_1 and c_2 balance the contributions of the Matérn and linear components of the model. This hybrid structure enhances the model's flexibility as it can fit nonlinear trends

within the range of known species, while still being capable of generalising linearly the unseen, extrapolated fossil inputs.

E.4. Log-Transformation of the Target. Since syllable rate values must be positive and spanned an order of magnitude, the target syllable rate was transformed as:

$$y_{log} = log\ (y)$$

This transformation improves normality, variance stability, and ensures that predicted rates remain positive. After prediction, values were exponentiated:

$$\hat{y} = exp\ (\mu \pm \sigma)$$

where μ is the mean predicted log-rate and σ is the standard deviation from the GPR posterior.

E.5. Hyperparameter Tuning via Bayesian Optimisation. Kernel hyperparameters, including $\ell, \nu, \sigma_0, c_1, c_2$ and noise level were optimised using Bayesian optimisation via the Optuna framework to efficiently explores complex, non-convex hyperparameter spaces (42). The objective function was defined as the negative mean squared error (MSE) over 3-fold cross-validation:

$$MSE = \frac{1}{n}\sum_{i=1}^{n}(y_i - \hat{y}_i)^2$$

The search for the parameters was terminated after 1000 trials or earlier if the best value did not improve for 200 consecutive iterations.

E.6. Model Evaluation. After training, model performance was evaluated on the full dataset using three metrics:

- Mean Absolute Error (MAE):

$$MAE = \frac{1}{n}\sum_{i=1}^{n}|y_i - \hat{y}_i|$$

Measures average absolute deviation, reflecting typical error magnitude.

- Root Mean Squared Error (RMSE):

$$RMSE = \sqrt{\frac{1}{n}\sum_{i=1}^{n}(y_i - \hat{y}_i)^2}$$

Emphasises larger errors due to squaring, useful for detecting outlier influence.

- Coefficient of Determination (R^2):

$$R^2 = 1 - \frac{\sum(y_i - \hat{y}_i)^2}{\sum(y_i - \bar{y})^2}$$

Represents the proportion of variance explained by the model. Values near 1 indicate high predictive accuracy.

All metrics were additionally normalised by the mean syllable rate to express relative performance.

E.7. Optimal hyper parameter. Following Bayesian optimisation, the Gaussian Process Regression model converged on the following kernel hyperparameters as optimal for predicting log-transformed syllable rates: $c_1 = 1.23$, $\ell = 1.46$, $\nu = 1.5$, $c_2 = 0.731$, $\sigma_0 = 0.136$, and $noise\ level = 5.95 \times 10^{-5}$. These values were used to construct the final kernel and train the model on the complete dataset.

E.8. Application to Fossil Data. The validated GPR model was applied to predict syllable rates in the fossil taxa. Each fossil species was represented by a vector of four inputs: stridulatory file length, tooth count, estimated calling frequency, and the estimated syllable duration. GPR outputs were converted from log space and plotted with standard deviation error bars to reflect prediction uncertainty.

For each fossil, the maximum possible rate was calculated to compare predicted values with the theoretical bound to ensure biological plausibility:

$$Max\ syllable\ rate = \frac{1000}{Syllable\ duration\ (ms)}$$

E.9. Model limitation. While GPR provides a powerful probabilistic framework for estimating syllable rates in extinct taxa, it is important to acknowledge the model's limitations in reconstructing full acoustic behaviours. The current model predicts syllable rate based on morphological and acoustic data under the assumption that these traits directly and consistently determine rhythmic sound production across taxa. However, this prediction represents only a single component of a more complex acoustic signal.

In particular, the model assumes that a species' call consists of simply regular, trill-like syllable production patterns. Real insect songs sometimes also include structured elements such as chirps, phrases, pauses, and modulations, shaped by neurophysiological controls, environmental pressures, and species-specific behavioural contexts which are not captured

by morphological features alone. Finally, some fossils may fall outside the range of known species used to train the model and should be interpreted with caution.

Still, the model's predicted syllable rates serve as a biomechanical estimate of sound rhythm potential, useful primarily for reconstructing hypothetical trilling signals under idealised conditions rather than complete reconstructions of fossil sound.

2. Supplemental Results

A. GPR model performance and evaluation. The trained Gaussian Process Regression (GPR) model demonstrated strong predictive performance when evaluated on the full dataset of extant insect species. The model was assessed using three standard regression metrics: mean absolute error (MAE), root mean squared error (RMSE), and the coefficient of determination (R^2). Predictions were made on the log-transformed syllable rate, then exponentiated to yield values in the original scale (syllables/second).

The mean absolute error (MAE) was 2.42 syllables/sec, corresponding to approximately 5.5% of the mean syllable rate in the dataset. This indicates that, on average, the model's predictions deviate from the true syllable rate by just over 2 syllables per second—a small discrepancy considering that some species reach rates over 90 syllables/sec. The root mean squared error (RMSE) was 4.10 syllables/sec (9.4%), showing that the model maintains good robustness even when occasional larger errors occur, and that extreme outliers are not a concern. The model achieved an R^2 value of 0.966, indicating that 96.6% of the variance in the observed syllable rates is explained by the GPR model. This level of explanatory power is considered excellent in biological data modelling and suggests that the chosen features—file length, tooth count, carrier frequency, and syllable duration—collectively contain strong predictive signals for acoustic output.

Visual inspection of the actual vs predicted plot (Fig. S9b) confirms that majority of predicted values fall close to the identity line (y = x), with no systematic over- or under-prediction across the range. Slight underestimation is observed for some of the highest syllable rates, which likely reflects the relatively lower representation of high-rate species in the training data and the conservative nature of GPR models when extrapolating near the boundaries of the input space.

Taken together, these results validate the use of Gaussian Process Regression for predicting insect syllable rates from morphological and acoustic features. The model's high accuracy, low error margins, and built-in uncertainty estimates make it well-suited for extrapolating to fossil specimens while maintaining biological plausibility.

B. Syllable rate prediction of fossilised insects. Predicted syllable rates for the nine fossil insect species varied widely across taxa, with corresponding confidence intervals reflecting the degree of uncertainty in each estimate (Fig S9c). Most predictions fell well below their respective biomechanical limits, which were computed as the inverse of syllable duration and served as hard upper bounds on calling rate.

Predicted syllable rates for nine fossil insect species using Gaussian Process Regression. Blue markers indicate the mean predicted syllable rate for each species, with vertical error bars representing standard deviation. Red markers denote the maximum syllable rate for each species.

Archaboilus polyneurus showed a moderate predicted rate ($33.72 \pm 9.64\ s^{-1}$), substantially below its theoretical maximum ($90.91\ s^{-1}$), indicating biologically plausible calling behaviour. In contrast, *Aboilus stratosus* exhibited a lower predicted rate ($8.39 \pm 2.42\ s^{-1}$), yet remained below its biomechanical ceiling ($25.32\ s^{-1}$).

Some taxa exhibited particularly low predicted syllable rates, including *Allaboilus gigantus* ($0.63 \pm 1.08\ s^{-1}$) and *Aboilinae sp. 1* ($3.26 \pm 2.50\ s^{-1}$), likely due to long syllable durations. These predictions, while conservative, were accompanied by relatively small standard deviations, indicating higher model confidence in the low estimates.

Other species such as *Sigmaboilus peregrinus* ($41.38 \pm 32.71\ s^{-1}$) and *Novaboilus ovatus* ($28.78 \pm 13.52\ s^{-1}$) showed wide predictive intervals, suggesting greater extrapolation uncertainty due to feature combinations not well represented in the extant training data. Nevertheless, their mean values remained comfortably below their duration-based limits (133.33 and $62.50\ s^{-1}$, respectively), upholding biological plausibility.

Across all species, predicted syllable rates remained below their corresponding physical maxima, and the distribution of values reflected both anatomical potential and statistical uncertainty.

3. Supplemental discussion

A. Notes on the FEA method. While we lack sufficient data of wing thickness across enough extant taxa to improve the method at this stage, we emphasize that this model was used only to recover the vibrational decay of the wing in response to stimulation in the time domain studies. It also allows for simpler modelling approaches to reduce computation times. The disadvantage is that if some species have unique membrane or vein thicknesses within the vibration region that modify the wing vibration, these are not captured in the model (43).

B. Relic condition of tegminal stridulation. The relic condition for wing stridulation appears to have started with wings developing sclerotised pegs, randomly distributed on special wing veins, as observed in some cockroaches (44), Mantids (45), in some living Tettigoniidae (e.g. *Pholidoptera* spp. (46)), and in the living Prophalangopsidae, which have traces of vestigial networks of wing veins that exhibit non-functional stridulatory teeth (Fig. S7). Random files struck at the time would produce only broadband calls, as observed in females of many living Tettigoniidae species, which evolved multiple stridulatory veins for duet acoustic response (e.g. many Phaneriopterinae species Nickle & Carlysle (47), and in Pseudophyllinae, e.g. *Panoploscelis* spp. (48)). The evolution of a single stridulatory file, with systematically arranged teeth, is a clear departure or improvement from the relic condition of stridulation.

C. Wing symmetry and stridulation. Most male Tettigoniidae have asymmetric wings, and have adopted left-over right-wing position, with the left wing bearing the active file, and the right wing the active plectrum. Yet, the earliest Tettigoniidae diverging lineage, the Nedubini, which exhibit symmetric wings, is ambidexterous (49), just like grigs (see below). On the other hand, males of Prophalangopsidae (the grigs, a relic family closely related to modern-day katydids), use symmetric wings and can switch wing overlap during stridulation, just like the earliest diverging Tettigoniidae lineage. Producing pure-tone low frequencies <10 kHz, requires two wings (or two speakers) oscillating in phase to maximise sound output and intensity(18) , and this is well studied in field crickets, which use pure tones at ca 5 kHz. The grig *Tarragilus diuturnus* uses similar frequencies, and to the human hear, their calls sound exactly like those of field crickets (Fig. S1). This suggests these species, and their extinct ancestors singing at frequencies below 10 kHz (20), used a wing coupling mechanism (like the field cricket escapement) to synchronise both wings in

vibration, and avoid destructive interference to maintain a pure-tone signal (18). Indication of this symmetrical mechanism could be identified in fossilised wings even if only one wing is found (see Fig. S5). For example, a single wing with a fully developed file and a fully developed plectrum (designed to scrape a functional file in the opposite wing), suggests that its counterpart likely had the same structures (symmetry) for ambidextrous stridulation.

D. Bimodal temporal/frequency modulation. A single wing closing phase produces the most basic oscillation of the call, termed here a ‘syllable’ (Movie 1). Syllables can be grouped or delivered in sequence to form a repertoire, which includes variations in time and frequency as the main parameters for specific recognition.

In many extant species, a systematic file-tooth distribution can affect the instantaneous frequency and amplitude components of the syllable. This has been described as mechanical frequency modulation (FM) and has been observed in the calls of the leaf mimicking katydids Pterochrozini (50). This seems to be also a mechanism adopted by some Jurassic Ensifera like *A. polyneurus* (see Fig. S6).

E. Insect ear evolution. Insect ears arise from the specialisation of pre-existing mechanoreceptor organs (precursor organs) that detect substrate vibrations into organs that detect airborne sound (51). Convincing support for a vibratory origin of the auditory system is given by many interneurons with bimodal vibratory and auditory response properties (52). Therefore, the ability to detect airborne sounds seems to be an evolutionary addition to the pre-existing adaptation (53). Importantly, the auditory sensilla precursor to the *cristica acustica* (CA) is found in the complex tibial scolopidial organ of atympanate Ensifera, where the sensilla are specialised in substrate vibration detection. Notable structural changes occurred during the transformation of scolopidial cells specialised in vibrations (low frequencies) to functional sound detectors.

A precursor scolopidium might have started being sensitive to high-amplitude sound before tympana and acoustic trachea appeared. A good example of this condition is preserved in the subgenual organ of some living cockroach species, and the high wing chordotonal organ of locust, both of which have been shown to respond to sound without any tympanal adaptation (54, 55). In this context, sound-capturing structures evolved in successive steps, transforming the cuticle into tympana, and a respiratory trachea into an air space to enhance

tympanal vibrations. Subsequently, the tracheal air space backing the tympana was modified into an acoustic trachea or ear canal (Fig. 5A-C, main text), which in modern katydid species focuses on capturing and conducting sounds to the tympanal organ. This gradual transition from vibrational to airborne hearing has been convergently followed in the mammalian transition from jaw bones to the formation of the middle ear ossicles (56). The timing of this mammalian hearing transition coincides with the specialisation of insectivorous dentition, suggesting that eavesdropping on and predation of singing insects were likely among the driving forces of this transition (57). The elongation of the cochlear canal to the cochlea of therian mammals was likely constrained and triggered by the coevolution between these small animals and their prey, acoustically active orthopterans (58).

F. Relic condition of the acoustic trachea. A bifurcated auditory trachea seems to be the plesiomorphic condition of a functional acoustic trachea, as it also occurs in atympanate Ensifera (e.g., Gryllacrididae, Fig. S8AB), and is also observed in nymphs during the ontogenetic development of Tettigoniidae species.

Ears have evolved at least 19 times independently in insects. In the period before bats, ears and complex acoustical behaviours appeared independently in at least seven insect orders (53, 59, 60). It was recently shown that at least four of the species-rich moth clades (Drepanoidea, Geometroidea, Noctuoidea, and Pyraloidea) evolved ears in the Late Cretaceous (some 103.4 to 67 Ma), several million years before the echolocating bat (61). The authors suggest that hearing organs in Lepidoptera, and in animals more broadly, evolved to provide auditory surveillance of the environment for broadband sounds produced by potential predators. Behavioural and neural evidence have shown that moths respond to the low-frequency (<20 kHz) walking and wingbeat sounds of predatory birds (62, 63). This suggests that the hearing organs of moths and other insects likely evolved first for auditory surveillance before subsequently being co-opted for bat detection.

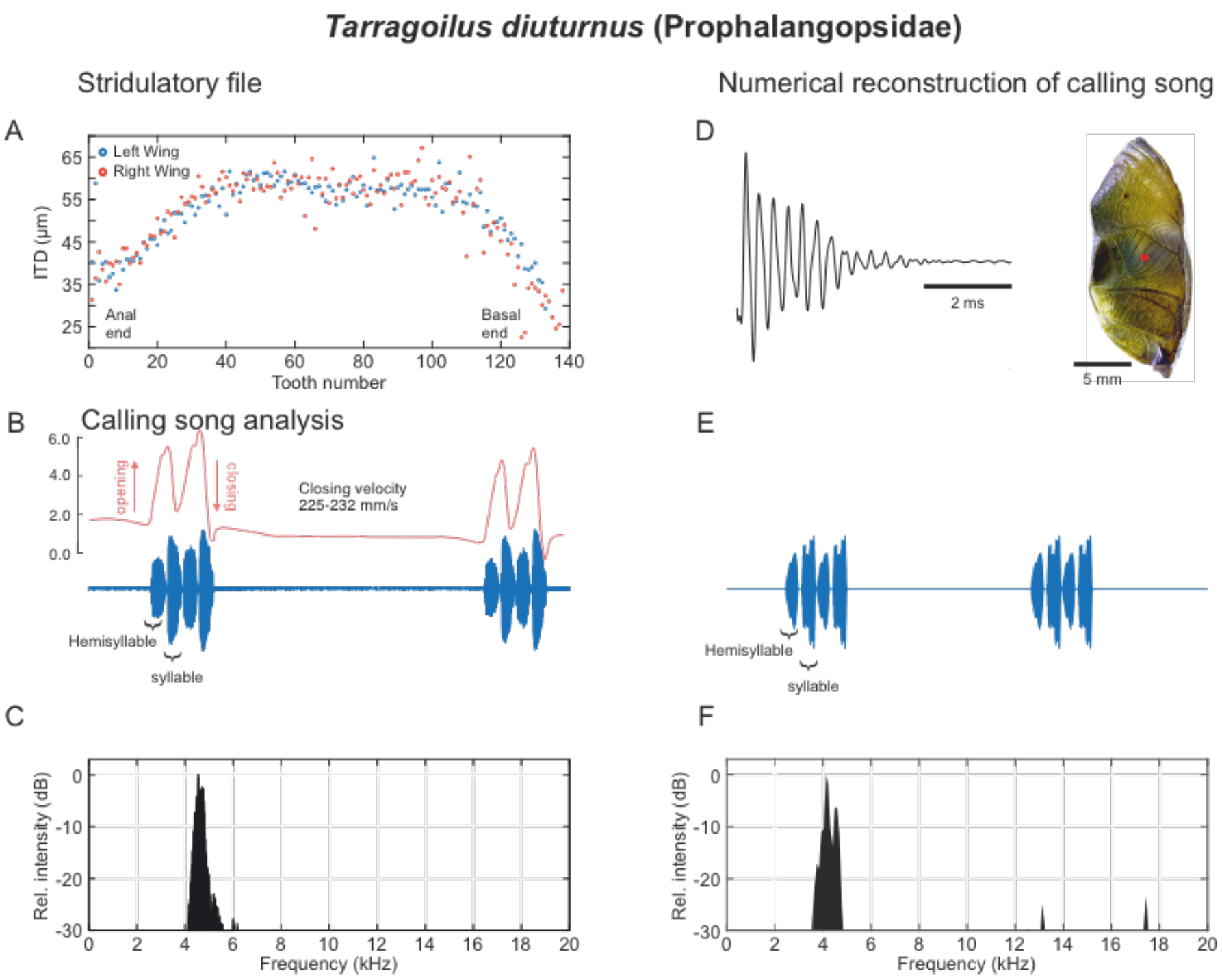

Fig. S1. *Tarragoilus diuturnus* (Prophalangopsidae) stridulatory file morphology, calling song and proof-of-concept numerical call reconstruction. (*A*) Tooth distribution on left- and right-wing files (blue and red circles, respectively) of one *T. diuturnus* male. (*B*) Two consecutive calling song chirps (blue oscillogram trace), each containing two syllables. The red line shows the wing motion associated with each syllable. Note that low-amplitude syllables are produced during the opening wing phase, and high-amplitude syllables during the closing phase. (*C*) Power spectrum of the calling song shown in (B). (*D*) Basic oscillation produced by a single tooth-plectrum click on a left wing of *T. diuturnus*. Vibrations were obtained by positioning the LDV laser (red dot) on the main resonant area of the wing while actuating the file using a dissected plectrum of the opposite wing. (*E*) Two consecutive chirps numerically recreated using information from file morphology and tooth-plectrum click shown in (D). (*F*) Power spectrum of the call shown in (E). disp. = displacement; ITD = inter-tooth-distance.

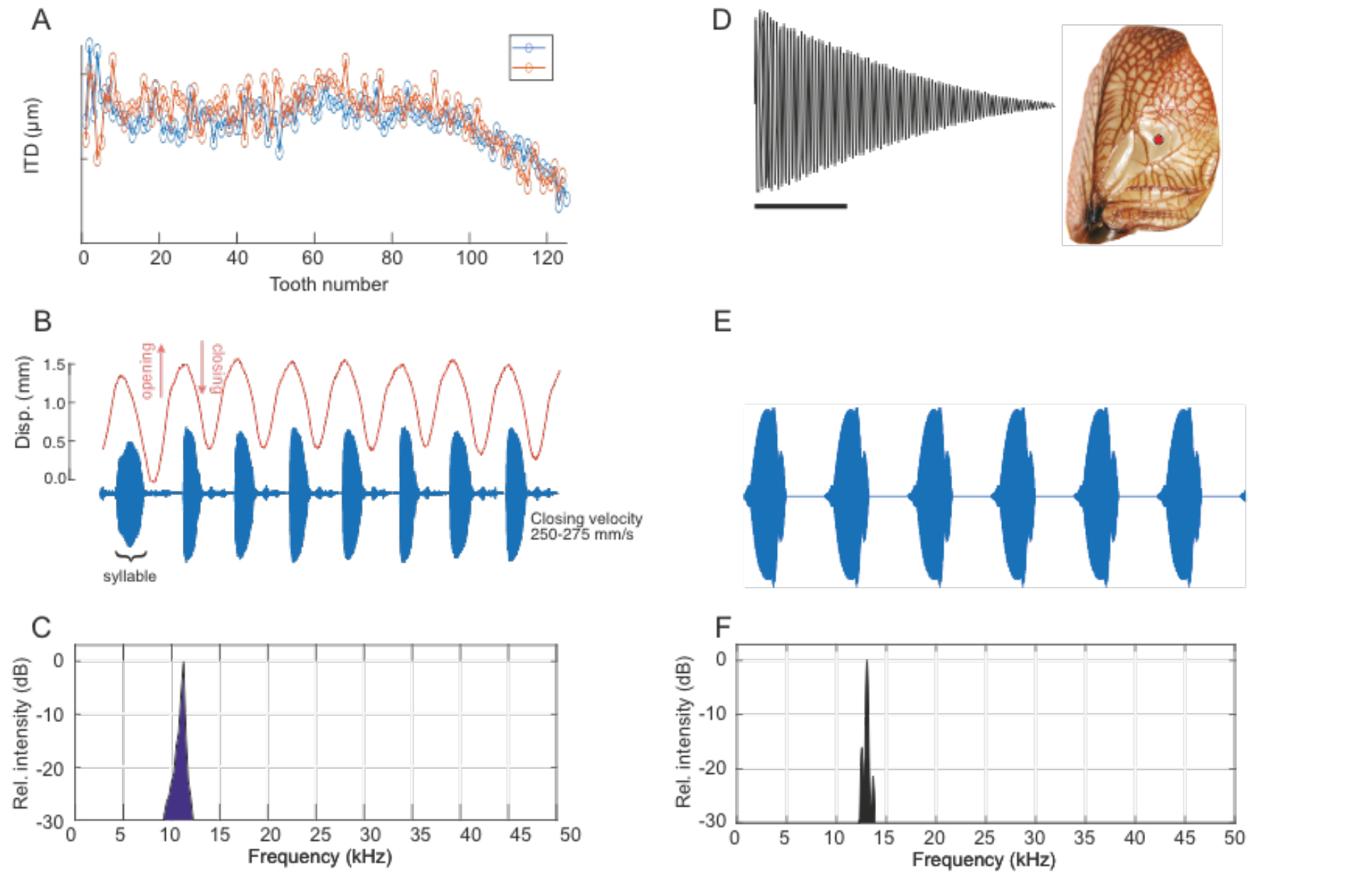

Fig. S2. *Cyphoderris monstrosa* (Prophalangopsidae) stridulatory file morphology, calling song and proof-of-concept numerical call reconstruction. **(*A*)** Tooth distribution on left- and right-wing files (blue and red circles, respectively) of one male. **(*B*)** Two consecutive calling song sequence(blue oscillogram trace), each containing two syllables. The red line shows the wing motion associated with each syllable. Note that low-amplitude syllables are produced during the opening wing phase, and high-amplitude syllables during the closing phase. **(*C*)** Power spectrum of the calling song shown in (B). **(*D*)** Basic oscillation produced by a single tooth-plectrum click on a right wing of a male. Vibrations were obtained by positioning the LDV laser (red dot) on the main resonant area of the wing while actuating the file using a dissected plectrum of the opposite wing. **(E)** Six consecutive calls numerically recreated using information from file morphology and tooth-plectrum click shown in (D). **(F)** Power spectrum of the call shown in (E). disp. = displacement; ITD = inter-tooth-distance.

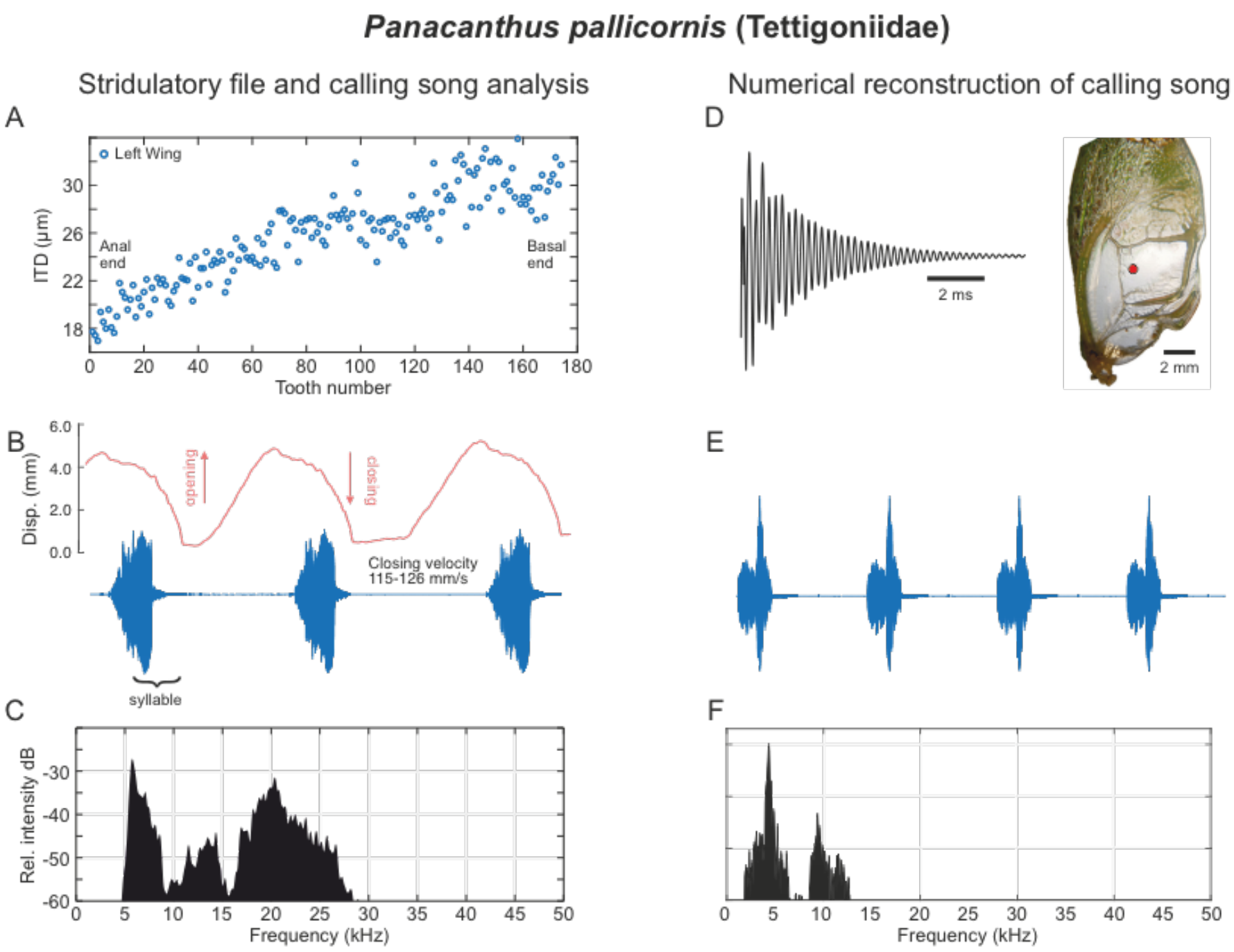

Fig. S3. *Panacanthus pallicornis* (Tettigoniidae) stridulatory file morphology, calling song and proof-of-concept numerical call reconstruction. (*A*) Tooth distribution on left (functional) file of one *P. pallicornis* male. (*B*) Three consecutive syllables (blue oscillogram trace) and associated wing motion (red line). Signals are produced during the wing-closing movement; opening of the wing is mostly silent. (*C*) Power spectrum of the syllables in (B). (*D*) Basic oscillation (left) produced by a single tooth-plectrum click on a right wing (right) of *P. pallicornis*. Vibrations were obtained by positioning the LDV laser (red dot) on the main resonant area of the wing while actuating the file using a dissected plectrum of the opposite wing. (*E*) Reconstructed syllable. (*F*) Power spectrum of the artificial call shown in (E). disp. = displacement; ITD = inter-tooth-distance.

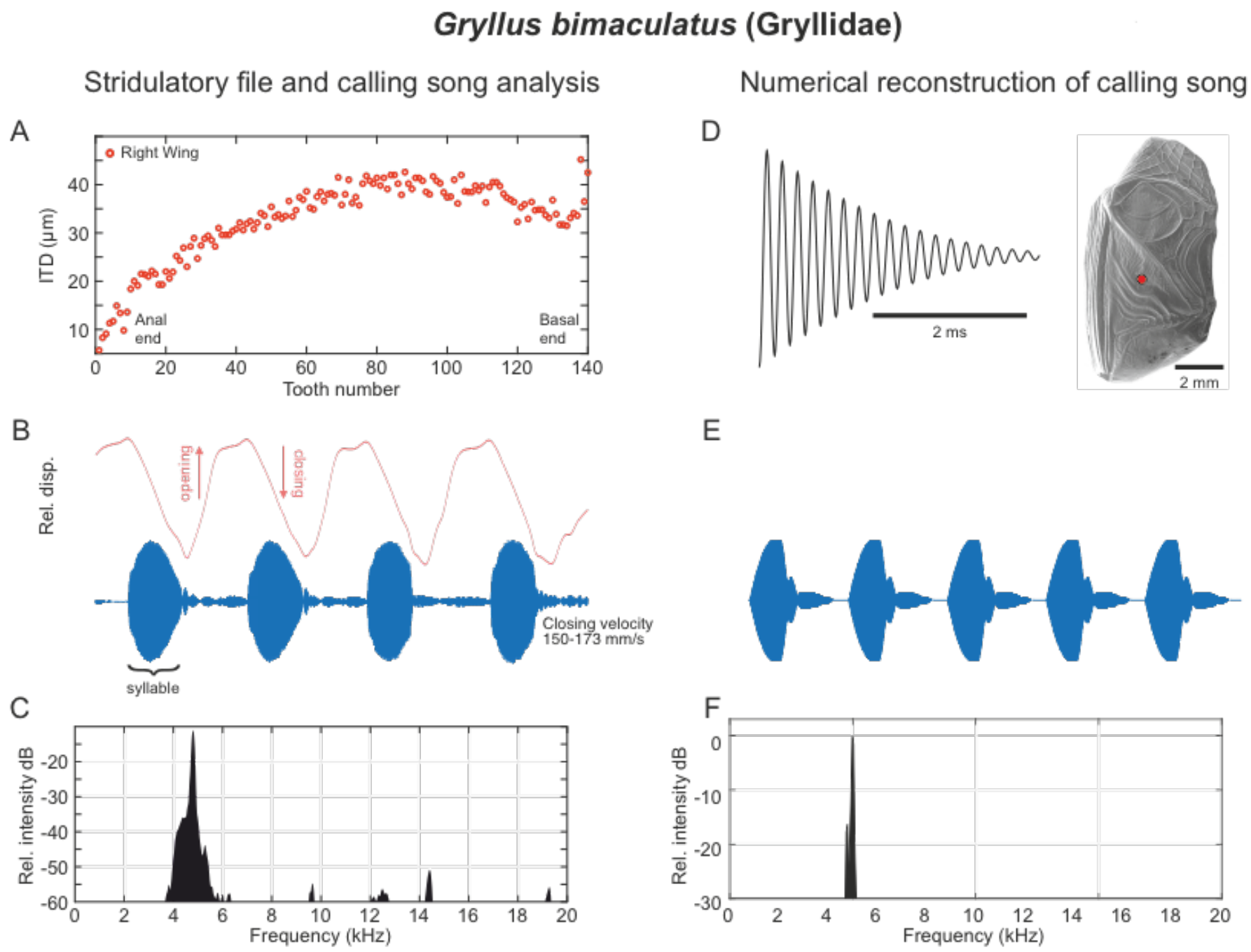

Fig. S4. *Gryllus bimaculatus* (Gryllidae) stridulatory file, calling song and proof-of-concept numerical call reconstruction. (*A*) Tooth distribution of right (functional) file of one *G. bimaculatus* male. (*B*) One chirp, containing four syllables (blue oscillogram trace) and associated wing motion (red line). Signals are produced during the wing-closing movement. (*C*) Power spectrum of the syllables in (B). (*D*) Basic oscillation (left) produced by a single tooth-plectrum click on a right wing (right) of *G. bimaculatus*. Vibrations were obtained by positioning the LDV laser (red dot) on the main resonant area of the wing while actuating the file using a dissected plectrum of the opposite wing. (*E*) Reconstructed chirp containing four syllables. (*F*) Power spectrum of the artificial call shown in (E). disp. = displacement; ITD = inter-tooth-distance.

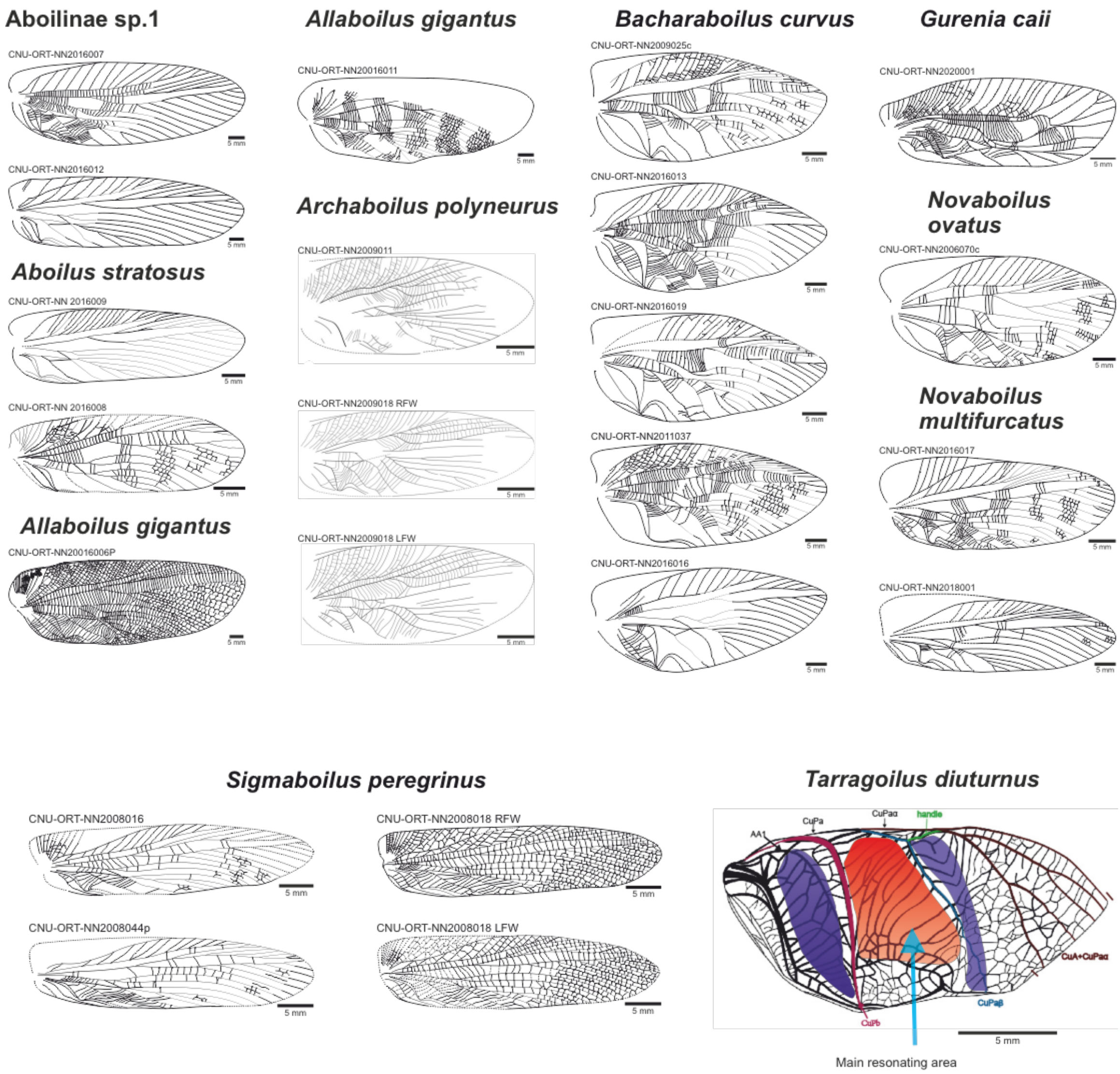

Fig. S5. Line drawings of male Prophalangopsidae and Haglidae forewing fossils used in this study. A line drawing of the right wing of *T. diuturnus* (Prophalangopsidae) is presented here for comparison purposes.

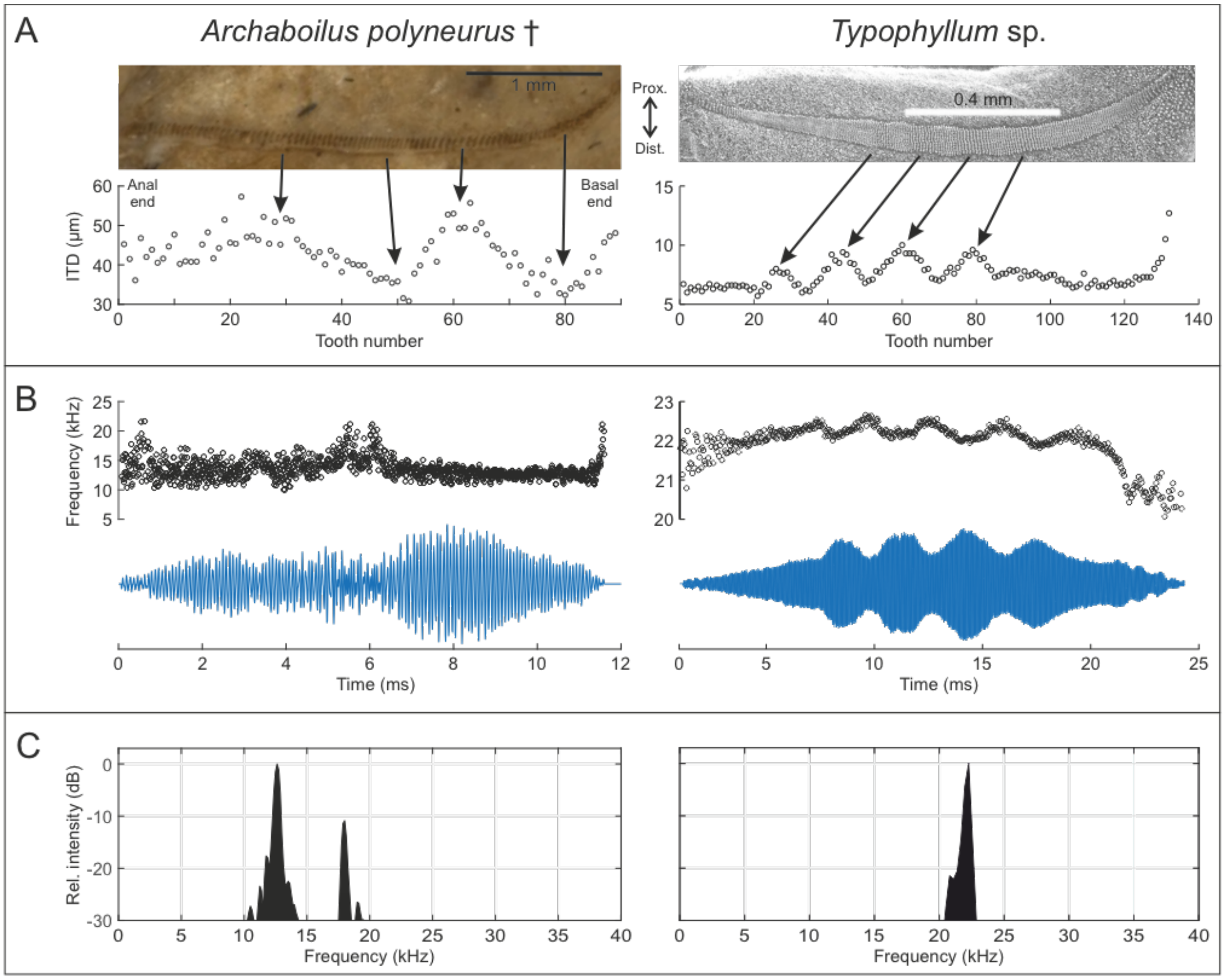

Fig. S6. Frequency modulation produced by file morphology in extinct (†) and living katydids: *Archaboilus polyneurus* (Haglidae, left) and *Typophyllum* sp. (Tettigoniidae, Pterochrozinae, right). (*A*) Stridulatory file morphology (top) and inter-tooth-distances (ITD; bottom). Arrows highlight areas of high ITDs. Prox.: proximal; Dist.: distal. (*B*) One syllable (blue oscillogram trace; bottom) created during the closing movement of the wings and thus the striking of subsequent teeth from anal to basal. Top trace: Zero-crossing frequency analysis of the syllables below. Note the effect of tooth arrangement on the instantaneous frequency (and, especially in *Typophyllum*, the amplitude) of the syllable. (*C*) Power spectra of the syllables in (B).

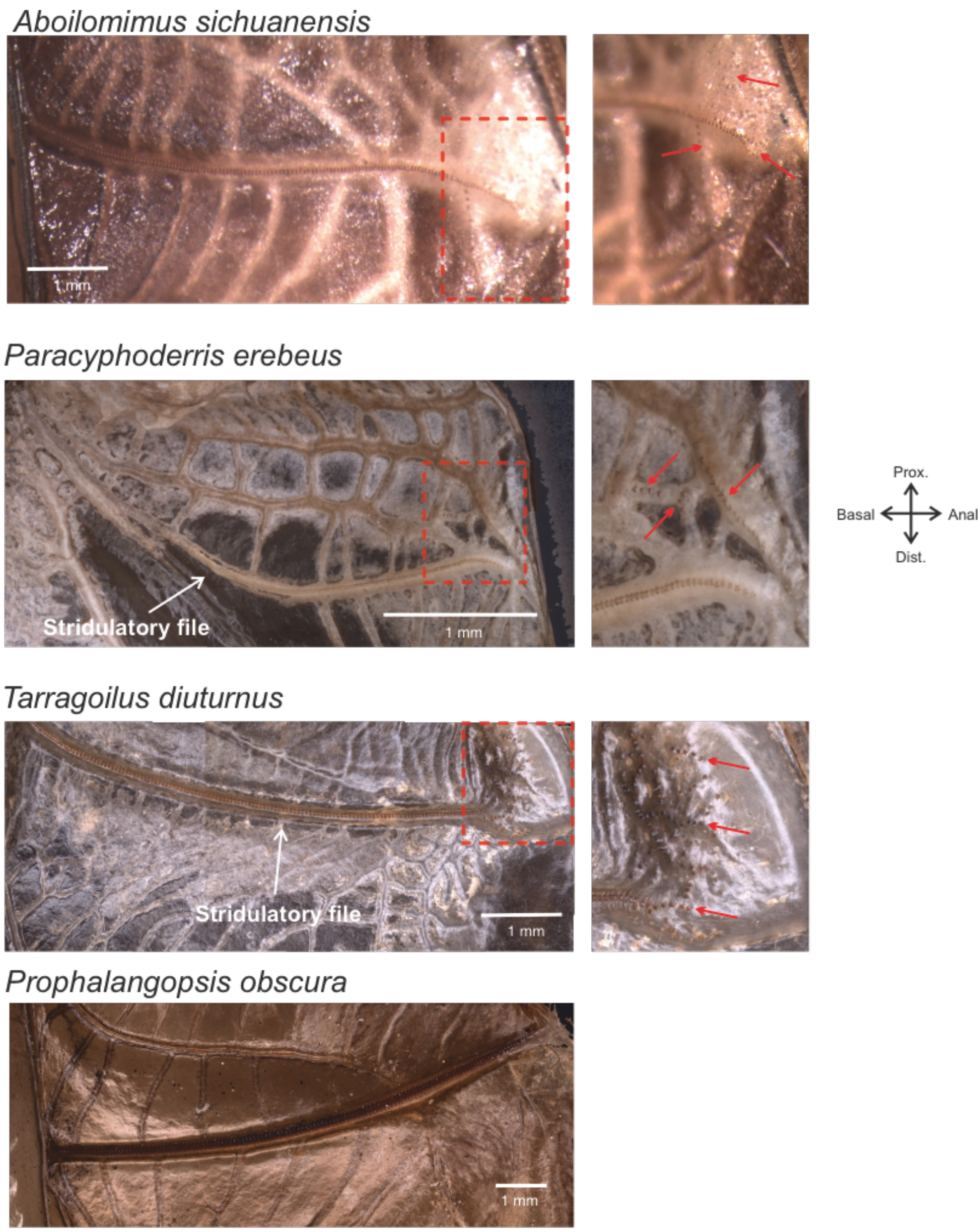

Fig. S7. The stridulatory region of three living Prophalangopsidae (from top to bottom: *Aboilomimus sichuanensis*, *Paracyphoderris erebeus*, *Tarragoilus diuturnus*) showing the stridulatory file (right) and adjacent veins with remains of non-functional pegs (right, red arrows). This evidences the potential plesiomorphic condition of stridulation using multiple files and scraping surfaces. Dashed rectangles indicate magnified regions. Dist.: distal; Prox.: proximal.

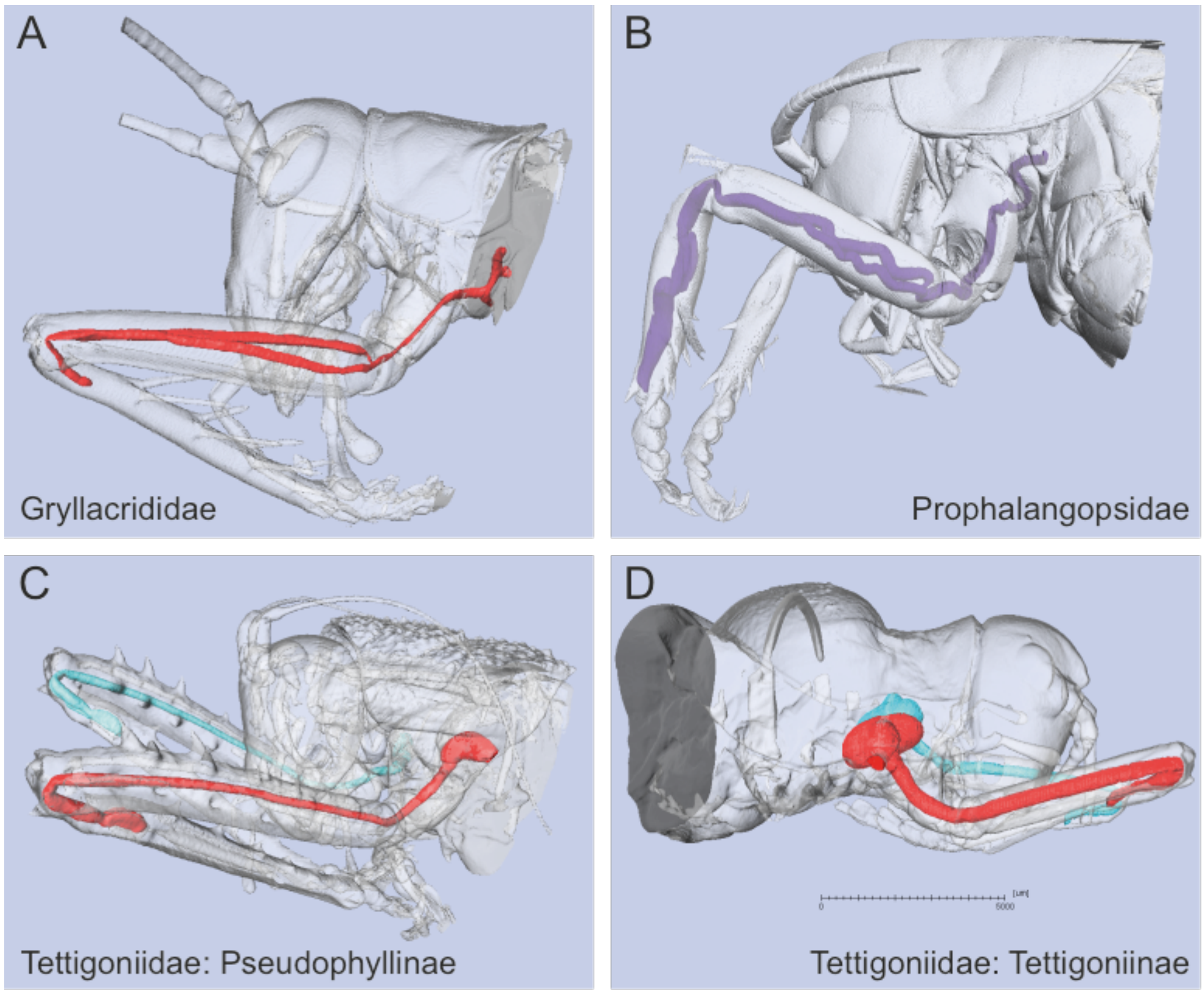

Fig. S8. The foreleg trachea and its different functions. (*A*) A bifurcated trachea in a species of Gryllacrididae *(Magnumtergalis* sp.), an atympanate family without known acoustic communication. (*B*) A bifurcated trachea in a living (tympanate) Prophalangopsidae *Tarragoilus diuturnus*. This trachea is unspecialised for sound capture, transmission or amplification. (*C-D*) Different forms of acoustic tracheae (or ear canals) in two Tettigoniidae subfamilies (c) Choeroparnops fulvus (Pseudophyllinae), and (d) *Phymonotus jacintotops* (Tettgoniinae) showing two different forms acoustic trachea.

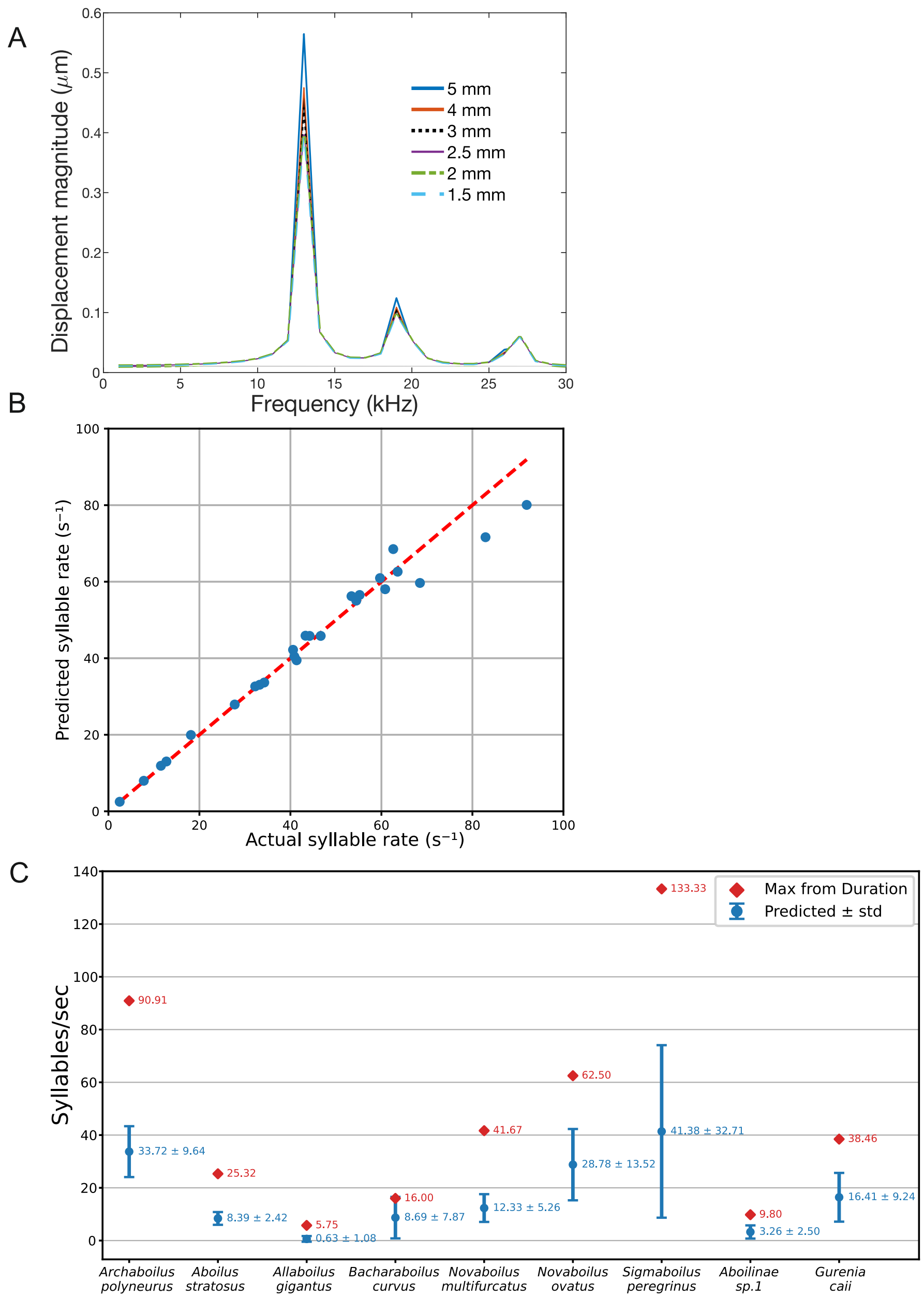

Fig. S9. (*A*) The mesh sensitivity analysis results for *Archaboilus polyneurus*. (*B*) : Actual vs Predicted syllable rates from Gaussian Process Regression. The dashed red line indicates the ideal fit (y = x). (*C*) Predicted syllable rates for nine fossil insect species using Gaussian Process Regression. Blue markers indicate the mean predicted syllable rate for each species, with vertical error bars representing standard deviation. Red markers denote the maximum syllable rate for each species.

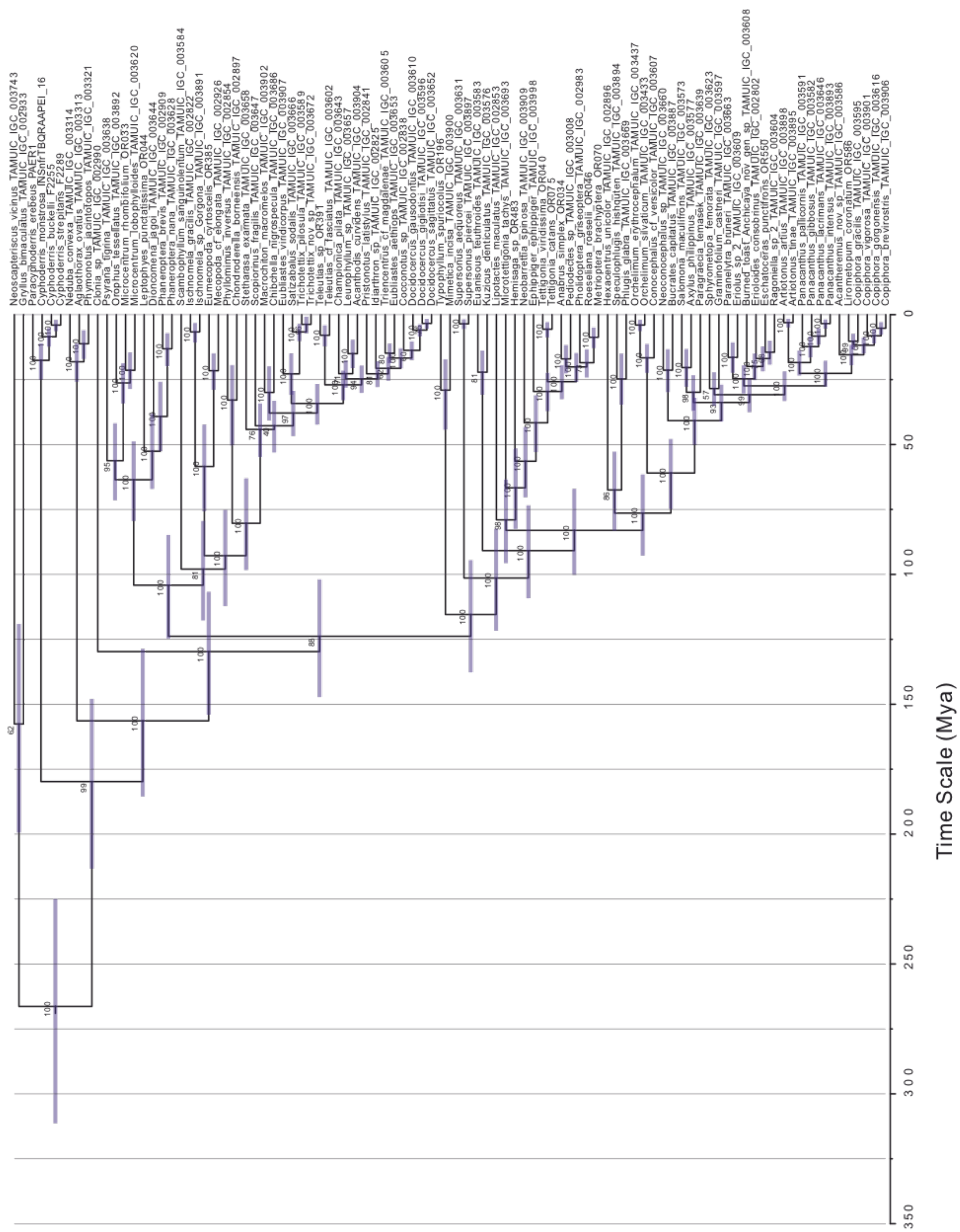

Fig. S10. Time-calibrated phylogeny of Ensifera from divergence-time estimation. Node ages are posterior means estimated from combined post–burn-in samples of converged MCMCtree chains, with blue bars showing 95% HPD credible intervals (Mya). Maximum-likelihood bootstrap support values are overlaid on the corresponding nodes. The full taxon set was dated to maximize temporal inference, then the tree was pruned to the subset of taxa with acoustic data for downstream comparative analyses.

Tables

Table S1. Raw data matrix of species, morphological data and carrier frequecny used in the PGLM analysis.

	Species	File length (mm)	Vibrating area (mm^2)	Frequency (kHz)		Ln file	Ln Vibrating Area	Ln Frequency
1	Acantheremus nov sp TAMUIC IGC 003586	1.43	2.80	24.00		0.36	1.02961942	3.17805383
2	Acanthodis curvidens TAMUIC IGC 003904	2.60	8.37	22.00		0.96	2.12465388	3.09104245
3	Aglaothorax ovatus TAMUIC IGC 003313	5.10	6.90	14.30		1.63	1.93152141	2.66025954
4	Anabrus simplex OR034	4.20	5.70	13.55		1.44	1.74046617	2.60638655
5	Artiotonus artius TAMUIC IGC 003898	1.75	1.58	40.70		0.56	0.45742485	3.70622809
6	Artiotonus tinae TAMUIC IGC 003895	1.70	1.24	35.40		0.53	0.21511138	3.56671182
7	Axylus philippinus TAMUIC IGC 003577	1.56	1.15	34.70		0.44	0.13976194	3.54673969
8	Bucrates capitatus TAMUIC IGC 003887	3.20	12.20	9.50		1.16	2.50143595	2.2512918
9	Burned toast Anchicaya nov gen sp TAMUIC IGC 003608	1.43	1.41	25.40		0.36	0.3435897	3.23474917
10	Championica pilata TAMUIC IGC 003643	1.97	2.38	22.70		0.68	0.86710049	3.12236492
11	Chibchella nigrospecula TAMUIC IGC 003686	3.04	10.34	9.42		1.11	2.33601987	2.24283509
12	Chondroderella borneensis TAMUIC IGC 002897	2.60	6.51	11.60		0.96	1.87333946	2.4510051
13	Clonia sp TAMUIC IGC 002990	2.46	2.88	18.00		0.9	1.05779029	2.89037176
14	Cocconotus sp TAMUIC IGC 002838	1.58	2.33	31.10		0.46	0.84586827	3.43720782
15	Conocephalus cf versicolor TAMUIC IGC 003607	1.25	0.97	36.00		0.22	-0.0304592	3.58351894
16	Copiphora brevirostris TAMUIC IGC 003906	3.20	4.46	15.60		1.16	1.49514877	2.74727091
17	Copiphora gorgonensis TAMUIC IGC 003616	1.98	2.49	22.60		0.68	0.91228271	3.11794991
18	Copiphora gracilis TAMUIC IGC 003595	2.00	3.65	20.60		0.69	1.29472717	3.02529108
19	Copiphora vigorosa TAMUIC IGC 003901	3.12	6.00	10.19		1.14	1.79175947	2.32140685
20	Cyphoderris buckelli F2255	2.85	3.30	13.30		1.05	1.19392247	2.58776404
21	Cyphoderris monstrosa INSnfrTBQRAAPEI 16	3.14	9.76	13.00		1.14	2.2782924	2.56494936
22	Cyphoderris strepitans F2289	3.30	9.07	12.70		1.19	2.20497226	2.54160199
23	Dioncomena jagoi TAMUIC IGC 003644	1.31	1.60	53.00		0.27	0.47000363	3.97029191
24	Docidocercus gausodontus TAMUIC IGC 003610	2.10	3.00	22.50		0.74	1.09861229	3.11351531
25	Docidocercus gigliotisi TAMUIC IGC 003596	2.41	2.73	26.80		0.88	1.00430161	3.28840189
26	Docidocercus sagittatus TAMUIC IGC 003652	2.00	2.90	30.00		0.69	1.06471074	3.40119738
27	Ephippiger ephippiger TAMUIC IGC 003998	4.30	5.93	20.00		1.46	1.78002421	2.99573227
28	Erioloides consobrinus TAMUIC IGC 002802	1.90	2.90	22.90		0.64	1.06471074	3.13113691
29	Eriolus sp 2 TAMUIC IGC 003609	1.74	1.35	30.30		0.55	0.30010459	3.41114771
30	Eschatoceras punctifrons OR550	0.74	0.54	64.00		-0.3	-0.6161861	4.15888308
31	Euanisous teuthroides TAMUIC IGC 003583	0.73	0.45	30.30		-0.31	-0.7985077	3.41114771
32	Eubliastes aethiops TAMUIC IGC 003653	2.87	3.76	21.60		1.05	1.32441896	3.07269331
33	Eubliastes viridicorpus TAMUIC IGC 003907	3.60	9.17	23.40		1.28	2.21593729	3.15273602
34	Eumecopoda cyrtoscelis OR385	2.63	5.60	3.10		0.97	1.7227666	1.13140211
35	Graminofolium castneri TAMUIC IGC 003597	1.45	0.85	27.70		0.37	-0.1625189	3.32143241
36	Gryllus bimaculatus TAMUIC IGC 002933	5.60	28.60	4.80		1.72	3.35340672	1.56861592
37	Hemisaga sp OR483	3.00	1.97	7.40		1.1	0.67803354	2.00148
38	Hexacentrus unicolor TAMUIC IGC 002896	2.10	8.80	11.00		0.74	2.17475172	2.39789527
39	Idiarthron sp TAMUIC IGC 002825	2.00	3.61	23.90		0.69	1.28370777	3.17387846
40	Ischnomela gracilis TAMUIC IGC 002822	2.50	1.98	15.10		0.92	0.68309684	2.71469474
41	Ischnomela sp Gorgona TAMUIC IGC 003891	2.46	1.95	16.80		0.9	0.66782937	2.82137889
42	Kuzicus denticulatus TAMUIC IGC 003576	1.10	1.46	39.60		0.1	0.37843644	3.67882912
43	Leptophyes punctatissima OR044	1.25	1.40	41.00		0.22	0.33647224	3.71357207
44	Leurophyllum sp TAMUIC IGC 003657	1.60	4.50	23.00		0.47	1.5040774	3.13549422
45	Lipotactes maculatus TAMUIC IGC 002853	1.20	1.50	33.10		0.18	0.40546511	3.49953328
46	Lirometopum coronatum OR586	3.20	11.40	10.80		1.16	2.43361336	2.37954613
47	Macrochiton macromelos TAMUIC IGC 003902	2.84	6.65	13.80		1.04	1.89461685	2.62466859
48	Mecopoda cf elongata TAMUIC IGC 002926	4.88	20.95	7.00		1.59	3.04213865	1.94591015
49	Metrioptera brachyptera OR070	1.32	2.54	30.00		0.28	0.93216408	3.40119738
50	Microcentrum lobophylloides TAMUIC IGC 003620	3.73	3.37	11.70		1.32	1.21491274	2.45958884
51	Microcentrum rhombifolium OR033	4.70	11.70	9.80		1.55	2.45958884	2.28238239
52	Microtettigonia tachys TAMUIC IGC 003693	0.70	0.75	50.00		-0.36	-0.2876821	3.91202301
53	Mimetica incisa TAMUIC IGC 003900	3.00	5.22	17.00		1.1	1.6524974	2.83321334
54	Neduba convexa TAMUIC IGC 003314	3.50	7.83	13.90		1.25	2.05796251	2.63188884
55	Neobarrettia spinosa TAMUIC IGC 003909	4.75	9.09	10.00		1.56	2.20717491	2.30258509
56	Neoconocephalus sp TAMUIC IGC 003660	3.40	5.16	12.00		1.22	1.64093658	2.48490665
57	Neoscapteriscus vicinus TAMUIC IGC 003743	2.18	7.20	3.17		0.78	1.97408103	1.15373159
58	Orchelimum erythrocephalum TAMUIC IGC 003437	2.90	6.70	13.60		1.06	1.90210753	2.61006979
59	Orchelimum silvaticum TAMUIC IGC 003433	2.52	5.20	18.00		0.92	1.64865863	2.89037176
60	Orophus tessellatus TAMUIC IGC 003892	2.59	2.43	10.50		0.95	0.88789126	2.35137526
61	Panacanthus gibbosus TAMUIC IGC 003582	5.20	19.35	6.40		1.65	2.96269242	1.85629799
62	Panacanthus intensus TAMUIC IGC 003893	4.48	12.74	11.50		1.5	2.54474665	2.44234704
63	Panacanthus lacrimans TAMUIC IGC 003646	5.43	16.27	7.13		1.69	2.78932292	1.96431123
64	Panacanthus pallicornis TAMUIC IGC 003591	6.27	14.16	4.70		1.84	2.65042109	1.54756251
65	Paracyphoderris erebeus PAER1	3.10	4.80	14.00		1.13	1.56861592	2.63905733
66	Paragraecia temasek TAMUIC IGC 003639	1.50	2.80	12.60		0.41	1.02961942	2.53369681
67	Paranelytra bruneri TAMUIC IGC 003663	1.03	0.94	40.90		0.03	-0.0618754	3.71113006
68	Pediodectes sp TAMUIC IGC 003008	2.60	1.30	18.00		0.96	0.26236426	2.89037176
69	Phaneroptera brevis TAMUIC IGC 002909	1.70	1.50	21.90		0.53	0.40546511	3.08648664
70	Phaneroptera nana TAMUIC IGC 003628	1.50	3.20	19.80		0.41	1.16315081	2.98568194
71	Phlugis glabra TAMUIC IGC 003669	0.46	0.56	49.50		-0.78	-0.5798185	3.90197267
72	Pholidoptera griseoptera TAMUIC IGC 002983	3.80	3.70	21.00		1.34	1.30833282	3.04452244
73	Phyllomimus inversus TAMUIC IGC 002854	4.40	9.90	10.00		1.48	2.29253476	2.30258509
74	Phymonotus jacintotopos TAMUIC IGC 003321	2.73	1.07	16.90		1	0.06765865	2.82731362
75	Pristonotus latistylus TAMUIC IGC 002841	5.10	20.22	10.00		1.63	3.00667221	2.30258509
76	Psyrana tigrina TAMUIC IGC 003638	2.20	1.10	35.50		0.79	0.09531018	3.5695327
77	Ragoniella sp 2 TAMUIC IGC 003604	2.10	4.99	15.80		0.74	1.60743591	2.76000994
78	Roeseliana roeselii OR046	1.56	3.70	21.00		0.44	1.30833282	3.04452244
79	Salomona maculifrons TAMUIC IGC 003573	2.27	2.33	30.50		0.82	0.84586827	3.41772668
80	Satizabalus sodalis TAMUIC IGC 003666	3.02	6.20	16.30		1.11	1.82454929	2.79116511
81	Scambophyllum sanguinolentum TAMUIC IGC 003584	1.50	2.23	23.70		0.41	0.80200159	3.16547505
82	Scopiorinus fragilis TAMUIC IGC 003647	2.26	21.21	25.60		0.82	3.05447277	3.24259235
83	Speculophlugis hishquten TAMUIC IGC 003894	0.51	0.29	50.00		-0.67	-1.2378744	3.91202301
84	Sphyrometopa femorata TAMUIC IGC 003623	3.28	4.58	11.00		1.19	1.521699	2.39789527
85	Stetharasa exarmata TAMUIC IGC 003658	2.90	4.12	22.85		1.06	1.41585316	3.12895112
86	Supersonus aequoreus TAMUIC IGC 003631	0.60	0.23	150.00		-0.51	-1.469676	5.01063529
87	Supersonus piercei TAMUIC IGC 003897	0.70	0.32	129.00		-0.36	-1.1394343	4.8598124
88	Teleutias cf fasciatus TAMUIC IGC 003602	1.20	2.14	24.10		0.18	0.76080583	3.18221184
89	Teleutias sp OR391 (cf castaneous)	1.40	1.52	42.10		0.34	0.41871033	3.74004774
90	Tettigonia catans OR075	2.50	5.90	8.00		0.92	1.77495235	2.07944154
91	Tettigonia viridissima OR040	2.40	4.00	12.03		0.88	1.38629436	2.48740353
92	Trichotettix nov sp TAMUIC IGC 003672	1.47	1.40	20.00		0.39	0.33647224	2.99573227
93	Trichotettix pilosula TAMUIC IGC 003589	1.71	1.39	16.90		0.54	0.32930375	2.82731362
94	Triencentrus cf magdalenae TAMUIC IGC 003605	2.79	3.80	21.00		1.03	1.33500107	3.04452244
95	Typophyllum spuriocolus OR196	5.50	17.30	7.00		1.7	2.8507065	1.94591015

Table S2. Predictions of carrier frequency for existing and newly presented fossil Ensifera, as well as two extant members of the Prophalangopsidae not included in the phylogeny, from OLS regressions (least informed approach), PGLS regressions (more informed approach), and phylogenetically informed predictions (most informed approach). Note that all previous predictions lie within the error range for the phylogenetically informed approach, while OLS and PGLS alone vary in their predictions.

Species	Family	File length	Node position	OLS	PGLS	Phylo-informed ± SE	Previous predictions	Reference
Archaboilus polyneurus †	Haglidae	4	98	10.56	7.38	9.75 ± 4.66	n/a	This study
Aboilus stratosus †	Prophalangopsidae	4	102	10.56	7.38	11.34 ± 5.20	n/a	This study
Allaboilus gigantus †	Prophalangopsidae	9.3	102	4.73	3.42	5.24 ± 2.41	n/a	This study
Bacharaboilus curvus †	Prophalangopsidae	9.2	102	4.78	3.46	5.30 ± 2.43	n/a	This study
Novaboilus multifurcatus †	Prophalangopsidae	5.1	102	8.38	5.91	9.08 ± 4.17	n/a	This study
Novaboilus ovatus †	Prophalangopsidae	8	102	5.46	3.93	6.02 ± 2.76	n/a	This study
Sigmaboilus peregrinus †	Prophalangopsidae	2.1	102	19.48	13.26	20.42 ± 9.37	n/a	This study
Aboilinae sp.1	Prophalangopsidae	7.2	102	6.04	4.32	6.62 ± 3.04	n/a	This study
Gurenia caii †	Haglidae	7.8	98	5.59	4.02	5.30 ± 2.53	n/a	This study
Eomortoniellus handlirschi †	Tettigoniidae	1.36	149	29.44	19.69	26.75 ± 8.71	32	Woodrow et al.[33]
Pseudotetigonia leona †	Tettigoniidae	4.2	106	10.08	7.06	11.19 ± 4.13	6.5	Greenwalt & Rust[5]
Archaboilus musicus †	Haglidae	9.34	98	4.71	3.41	4.49 ± 2.15	6.4	Gu et al.[4]
Pseudotetigonia amoena †	Tettigoniidae	4.2	106	10.08	7.06	11.19 ± 4.13	7.0	Rust et al.[6]
Prophalangopsis obscura	Prophalangopsidae	10.3	99	4.30	3.12	4.11 ± 2.00	4.7	Woodrow et al.[33]
Tarragoilus diuturnus	Prophalangopsidae	7.0	99	6.20	4.43	4.85 ± 2.79	4.6 (known)	This study

Table S3. Damping (ζ) and Quality factor (Q) measurement for the wings of living Ensifera specimens, using LDV.

	Gryllus bimaculatus	***Cyphoderris monstrosa***	***Tarragoilus diuturnus***	***Panacanthus pallicornis***	***Docidocercus sagittatus***
Wing resonant frequency	4.8 kHz *N=26*	13.5 kHz *N=8*	4.5 kHz *N=10*	5.0 kHz *N=3*	25.0 kHz *N=3*
LW ζ	0.0295696 ± 0.010328	0.036226 ± 0.007575	0.029570 ± 0.008066	0.081738777 ± 0.048247953	0.055495536 0.001196257
RW ζ	0.027596 ± 0.010586	0.041021 ± 0.016782	0.035604 ± 0.014615	0.03453006 ± 0.011626186	0.042616392 0.007486051
Q_{-3} LW	19.306923 ±7.675369	14.286165 ± 2.694056	19.084877 ± 5.992190	6.097554938± 4.988570281	9.012541632 0.195407467
Q_{-3} RW	20.423077 ± 6.587378	14.212860 ± 6.067572	17.903415 ± 11.819217	15.54069767± 4.788834981	11.98269936 2.146699289

Table S4. Wing membrane thickness in relation to eigenfrequency models and phylogenetically informed prediction of carrier frequency (fc) used in COMSOL models for each species. Note the FEA eigenfrequency is not a model prediction, but obtained by adjusting wing thickness until within the range of the phylogenetically informed prediction.

Species	Family	Wing thickness (μm)	FEA obtained eigenfrequency (kHz)	Phylo-informed fc (kHz ± SE)
Archaboilus polyneurus †	Haglidae	7.0	13.1	9.75 ± 4.66
Aboilus stratosus †	Prophalangopsidae	6.5	11.8	11.34 ± 5.20
Allaboilus gigantus †	Prophalangopsidae	18.0	8.8	5.24 ± 2.41
Bacharaboilus curvus †	Prophalangopsidae	10.0	5.0	5.30 ± 2.43
Novaboilus multifurcatus †	Prophalangopsidae	5.5	10.5	9.08 ± 4.17
Novaboilus ovatus †	Prophalangopsidae	6.5	5.6	6.02 ± 2.76
Sigmaboilus peregrinus †	Prophalangopsidae	7.0	20.5	20.42 ± 9.37
Aboilinae sp.1	Prophalangopsidae	10.0	6.0	6.62 ± 3.04
Gurenia caii †	Haglidae	7.0	5.9	5.30 ± 2.53

Table S5. Material properties implemented in the numerical models of the fossilised wings.

Wing Material Properties	Constant value	References
Young's Modulus (GPa)	9	Smith et al. [76]<br>Woodrow et al.[33]<br>Vincent & Wegst[138]
Density (kg/m^3)	1200	Vincent & Wegst[138]<br>Woodrow et al.[33]
Poisson's Ratio	0.33	Jafarpour et al.[139]<br>Woodrow et al.[33]

Table S6. Fossil calibrations used for divergence-time estimation in Ensifera. Fossils and nodal placements follow the conservative synapomorphy-based framework of Song et al. (2020), with calibrations shifted to deeper nodes when crown placement was uncertain. Two additional katydid fossils (Archepseudophylla spp. and Eomortoniellus handlirschi) were included to strengthen sampling within Tettigoniidae. Minimum ages reflect stratigraphic constraints, and soft maxima were implemented with broad skew-t priors, using the Rhynie Chert (412 Ma) as a conservative soft maximum for the root and selected deep nodes to accommodate fossil-record uncertainty.

Fossil	Species	Designation	Nodal Placement	Locality	Min Age (mya)	Max Age (mya)	Notes
1	Raphogla rubra	Oldest definitive Orthoptera	MRCA of Orthoptera / Root	France	283.8	412	See discussion
2	Protogryllus spp.	Stem Gryllidea	MRCA of Ensifera	Germany	182.9	412	See discussion
3	Pseudaboilus wealdensis	Stem-Tettigonoidea+Hagloidea	MRCA of Tettigoniidae + Prophalangopsidae + Stenopelmatoidea	United Kingdom	121.4	412	See discussion
4	Cratotetraspinus fossorius	crown-(Gryllotalpoidea+Grylloidea)	MRCA of Gryllidea	Brazil	113.2	412	See discussion
5	Archepseudophylla spp.	Crown Pseudophyllinae	MRCA of Asian Pseudophyllinae + Sister	Southwest China	33.9	412	clear synapomorphies with Pseudophyllinae, placed at node for monophyletic Pseudophylinae, not including the clade with Mecopodinae
6	Eomortoniellus handlirschi	Lipotactinae crown	MRCA of Lipotactinae	Baltic Region	33.9	412	clear synapomorphies with Lipotactinae

Table S7. Training dataset for Gaussian Process Regression model. Predictor variables include stridulatory file length (mm), total tooth number, dominant frequency (kHz), and syllable duration (ms). Syllable rate (syllables s^{-1}) was calculated at 20 °C using published temperature dependent relationships.

Species	Family	File length [mm]	Number of teeth	f_c [kHz]	Syllable duration [ms]	Syllable rate (s-1) at 20°C
Cyphoderris strepitans	Prophalangopsidae	3.1	88	12.7	4.1	43.32
Cyphoderris monstrosa	Prophalangopsidae	3.49	110	13	6.6	60.83
Cyphoderris buckelli	Prophalangopsidae	2.25	72	13.3	3.6	53.42
Cyrtoxopha gundlachi	Trigonidiinae	1.34	192	5.9	32	12.75
Cyrtoxopha columbiana	Trigonidiinae	1.29	150	6.6	17	27.76
Cyrtoxopha nola	Trigonidiinae	1.04	120	6.4	15.5	40.88
Cyrtoxopha confusa	Trigonidiinae	0.85	88	7.5	9.5	68.47
Neoconocephalus bivocatus	Tettigoniidae	2.5	83.6	9	3.5	59.69
Neoconocephalus triops	Tettigoniidae	2.1	75.8	11	10	91.93
Amblycorypha alexanderi	Tettigoniidae	2.19	97.7	13.6	113	2.48
Amblycorypha rotundifolia	Tettigoniidae	1.91	93	10	16	18.13
Amblycorypha bartrami	Tettigoniidae	1.99	92.94	9.76	40	7.78
Neoxabea bipunctata	Gryllidae	0.94	21.5	3.4	8	82.88
Anurogryllus arboreus	Gryllidae	2.4	72	5.6	7	63.57
Oecanthus celerinictus	Gryllidae	1.07	41.1	3.9	13	46.66
Oecanthus quadripunctatus	Gryllidae	1.47	56.7	4.2	16	32.26
Oecanthus exclamationis	Gryllidae	0.97	20.5	2.6	8	62.6
Oecanthus niveus	Gryllidae	1	26	3	13.5	54.48
Oecanthus fultoni	Gryllidae	1.79	41.3	2.9	16.2	40.58
Oecanthus latipennis	Gryllidae	1.48	42.1	2.9	19	41.36
Orocharis saltator	Gryllidae	2.08	77	5.1	15	44.22
Orocharis luteolira	Gryllidae	1.97	75	5.1	12	55.21
Orocharis tricornis	Gryllidae	2.16	120	6.1	19.5	33.24
Orocharis nigrifrons	Gryllidae	1.96	135	4.8	21	34.25
Orocharis diplastes	Gryllidae	1.95	104	5.7	25	11.54

Audio S1 (separate file). Reconstructed katydid ancestor calls from the Jurassic.

Movie S1 (separate file). High-speed video recording of a stridulating male Gryllus bimaculatus, captured at 10,000 frames per second. This footage offers a striking window into the mechanics of stridulation, revealing how the cricket shapes and controls its carrier frequency in real time. It's an invaluable visual tool for understanding both the precision and the complexity of acoustic signaling in this species.

Movie S2 (separate file). Wing-vibration animation of a male field cricket's forewing, generated through a numerical model using a simplified two-dimensional wing geometry. This models serve as a proof-of-concept, allowing us to compare its predictions with the real vibrational behavior of the same insect's wing, measured through Laser Doppler Vibrometry. The numerical model requires investigating the wing's material properties, which determines how faithfully the model can capture the true dynamics of the living structure.

Movie S3 (separate file). Wing-vibration animation of a fossilised forewing from the Jurassic ensiferan *Sigmaboilus peregrinus*, generated through a numerical model using the two-dimensional wing geometry approach described in Movie 2. The simulation uses material properties measured from living species, allowing us to explore how this ancient wing might have behaved in life and to draw tentative comparisons between fossil morphology and the vibrational mechanics of modern relatives.

Movie S4 (separate file). Reconstructed katydid ancestor calls from the Jurassic, matching a each species wing, used in the reconstruction, with the respective call obtained.

Movie S5 (separate file). Wing resonance of a male *Tarragoilus diuturnus*, one of the few relic living ensiferans, showing the main wing cells responsible for producing this species' characteristic ~5 kHz call. The wings were stimulated sympathetically using broadband sound, and their vibrational response was captured with a Micro-Scanning Laser Doppler Vibrometry. The result highlights the remarkable precision and acoustic specialization preserved in this ancient lineage.

Movie S6 (separate file). Measure of wing displacement amplitude of the right forewing of the relic living Ensifera, *Tarragoilus diuturnus*. The wings have been stimulated sympathetically with broadband sound, and recording using a Micro-Scanning Lasser Doppler Vibrometry, as explained for Movie 5. Profile dissecting lines show the displacement of the main wing area involved in sound radiation.

Software S1 (separate file). Matlab code for call reconstruction based on excitation of a fossilised wing recovered in 2d. Please consult DOI: 10.5281/zenodo.19882665 to access the code.

Software S2 (separate file). Code for ChatGPT (GPT-4o, OpenAI) used to assist in the development and implementation of the Python-based Gaussian Process Regression (GPR) framework and the Optuna-based hyperparameter optimization script. File name: GPR_Syllable_Rate_Model_v1 copy.txt. Read additional documentation (Licence.txt; Readme.txt; Requirement.txt).

SI References

1. A. M. Bolger, M. Lohse, B. Usadel, Trimmomatic: a flexible trimmer for Illumina sequence data. *Bioinformatics* **30**, 2114–2120 (2014).
2. R. Luo, *et al.*, SOAPdenovo2: an empirically improved memory-efficient short-read de novo assembler. *Gigascience* **1**, 2047-217X-1–18 (2012).
3. M. Petersen, *et al.*, Orthograph: a versatile tool for mapping coding nucleotide sequences to clusters of orthologous genes. *BMC Bioinformatics* **18**, 111 (2017).
4. S. Shin, *et al.*, Orthoptera-specific target enrichment (OR-TE) probes resolve relationships over broad phylogenetic scales. *Sci. Rep.* **14**, 21377 (2024).
5. K. Katoh, D. M. Standley, MAFFT Multiple Sequence Alignment Software Version 7: Improvements in Performance and Usability. *Mol. Biol. Evol.* **30**, 772–780 (2013).
6. S. G. Seabra, *et al.*, Different Genomic Changes Underlie Adaptive Evolution in Populations of Contrasting History. *Mol. Biol. Evol.* **35**, 549–563 (2018).
7. A. M. Kozlov, D. Darriba, T. Flouri, B. Morel, A. Stamatakis, RAxML-NG: a fast, scalable and user-friendly tool for maximum likelihood phylogenetic inference. *Bioinformatics* **35**, 4453–4455 (2019).
8. U. Mai, S. Mirarab, TreeShrink: fast and accurate detection of outlier long branches in collections of phylogenetic trees. *BMC Genomics* **19**, 272 (2018).
9. P. B. Frandsen, *et al.*, Phylogenomics recovers multiple origins of portable case making in caddisflies (Insecta: Trichoptera), nature's underwater architects. *Proceedings of the Royal Society B: Biological Sciences* **291**, 20240514 (2024).
10. M. L. Borowiec, AMAS: a fast tool for alignment manipulation and computing of summary statistics. *PeerJ* **4**, e1660 (2016).
11. B. Q. Minh, *et al.*, IQ-TREE 2: New Models and Efficient Methods for Phylogenetic Inference in the Genomic Era. *Mol. Biol. Evol.* **37**, 1530–1534 (2020).
12. S. Kalyaanamoorthy, B. Q. Minh, T. K. F. Wong, A. von Haeseler, L. S. Jermiin, ModelFinder: fast model selection for accurate phylogenetic estimates. *Nat. Methods* **14**, 587–589 (2017).

13. O. Chernomor, A. von Haeseler, B. Q. Minh, Terrace Aware Data Structure for Phylogenomic Inference from Supermatrices. *Syst. Biol.* **65**, 997–1008 (2016).
14. D. T. Hoang, O. Chernomor, A. von Haeseler, B. Q. Minh, L. S. Vinh, UFBoot2: Improving the Ultrafast Bootstrap Approximation. *Mol. Biol. Evol.* **35**, 518–522 (2018).
15. B. Hedwig, "A highly sensitive opto-electronic system for the measurement of movements" (2000).
16. F. Montealegre-Z, A. C. Mason, The mechanics of sound production in Panacanthus pallicornis (Orthoptera: Tettigoniidae: Conocephalinae): the stridulatory motor patterns. *Journal of Experimental Biology* **208**, 1219–1237 (2005).
17. B. Wenzel, B. Hedwig, Neurochemical control of cricket stridulation revealed by pharmacological microinjections into the brain. *Journal of Experimental Biology* **202**, 2203–2216 (1999).
18. T. Jonsson, F. Montealegre-Z, C. D. Soulsbury, D. Robert, Tenors Not Sopranos: Bio-Mechanical Constraints on Calling Song Frequencies in the Mediterranean Field-Cricket. *Front. Ecol. Evol.* **9**, 647786 (2021).
19. E. Celiker, *et al.*, Mechanical network equivalence between the katydid and mammalian inner ears. *PLoS Comput. Biol.* **20**, e1012641 (2024).
20. C. Woodrow, E. Baker, T. Jonsson, F. Montealegre-Z, Reviving the sound of a 150-year-old insect: The bioacoustics of Prophalangopsis obscura (Ensifera: Hagloidea). *PLoS One* **17**, e0270498 (2022).
21. B. D. Chivers, *et al.*, Functional morphology of tegmina-based stridulation in the relict species Cyphoderris monstrosa (Orthoptera: Ensifera: Prophalangopsidae). *Journal of Experimental Biology* **220**, 1112–1121 (2017).
22. G. K. Morris, R. E. Pipher, Tegminal amplifiers and spectrum consistencies in Conocephalus nigropleurum (Bruner), Tettigoniidae. *J. Insect Physiol.* **13**, 1075–1085 (1967).
23. F. Montealegre-Z, Scale effects and constraints for sound production in katydids (Orthoptera: Tettigoniidae): correlated evolution between morphology and signal parameters. *J. Evol. Biol.* **22**, 355–366 (2009).

24. F. Montealegre-Z, J. Ogden, T. Jonsson, C. D. Soulsbury, Morphological determinants of signal carrier frequency in katydids (Orthoptera): a comparative analysis using biophysical evidence of wing vibration. *J. Evol. Biol.* **30**, 2068–2078 (2017).
25. V. Godthi, R. Pratap, Dynamics of cricket sound production. *J. Vib. Acoust.* **137** (2015).
26. C. W. Smith, R. Herbert, R. J. Wootton, K. E. Evans, The Hind Wing of the Desert Locust (Schistocerca Gregaria Forskål): II. Mechanical Properties and Functioning of the Membrane. *Journal of Experimental Biology* **203**, 2933–2943 (2000).
27. COMSOL Multiphysics, *Structural Mechanics Module–User's Guide, Version 6.1* (COMSOL AB, 2022).
28. D. Veitch, *et al.*, A narrow ear canal reduces sound velocity to create additional acoustic inputs in a microscale insect ear. *Proc. Natl. Acad. Sci. U. S. A.* **118**, e2017281118 (2021).
29. C. E. Rasmussen, C. K. I. Williams, *Gaussian Processes for Machine Learning* (MIT Press, 2006).
30. J. J. Whitesell, T. J. Walker, Photoperiodically determined dimorphic calling songs in a katydid. *Nature* **274**, 887–888 (1978).
31. T. J. Walker, J. J. Whitesell, R. D. Alexander, The robust conehead: two widespread sibling species (Orthoptera: Tettigoniidae: Neoconocephalus 'robustus'). *Ohio Journal of Science* **73**, 321–330 (1973).
32. T. J. Walker, T. G. Forrest, J. D. Spooner, The rotundifolia complex of the genus Amblycorypha (Orthoptera: Tettigoniidae): songs reveal new species. *Ann. Entomol. Soc. Am.* **96**, 433–447 (2003).
33. T. J. Walker, The uhleri group of the genus Amblycorypha (Orthoptera: Tettigoniidae): extraordinarily complex songs and new species. *J. Orthoptera Res.* **13**, 169–183 (2004).
34. T. J. Walker, Systematics and acoustic behavior of United States crickets of the genus Cyrtoxipha (Orthoptera: Gryllidae). *Ann. Entomol. Soc. Am.* **62**, 945–952 (1969).
35. T. J. Walker, Systematics and acoustic behavior of United States crickets of the genus Orocharis (Orthoptera: Gryllidae). *Ann. Entomol. Soc. Am.* **62**, 752–762 (1969).
36. T. J. Walker, The taxonomy and calling songs of United States tree crickets (Orthoptera: Gryllidae: Oecanthinae). *Ann. Entomol. Soc. Am.* **55**, 303–322 (1962).

37. K. N. Prestwich, T. J. Walker, Energetics of singing in crickets: effect of temperature in three trilling species (Orthoptera: Gryllidae). *Journal of Comparative Physiology B* **143**, 199–212 (1981).
38. G. K. Morris, D. T. Gwynne, Geographical distribution and biological observations of Cyphoderris (Orthoptera: Haglidae) with a description of a new species. *Psyche (Stuttg).* **85**, 147–167 (1978).
39. C. R. Scotese, An atlas of Phanerozoic paleogeographic maps: the seas come in and the seas go out. *Annu. Rev. Earth Planet. Sci.* **49**, 669–718 (2021).
40. H. C. Jenkyns, L. Schouten-Huibers, S. Schouten, J. S. Sinninghe Damsté, Warm Middle Jurassic-Early Cretaceous high-latitude sea-surface temperatures from the Southern Ocean. *Climate of the Past* **8**, 215–225 (2012).
41. L. Chen, *et al.*, Reconstructing fossil insect communities and palaeoclimates for the Middle Jurassic Yanliao Biota in the Daohugou area of China. *Sci. Bull. (Beijing).* (2024). https://doi.org/10.1016/j.scib.2024.11.010.
42. T. Akiba, S. Sano, T. Yanase, T. Ohta, M. Koyama, Optuna: A Next-generation Hyperparameter Optimization Framework. *Proceedings of the ACM SIGKDD International Conference on Knowledge Discovery and Data Mining* 2623–2631 (2019). https://doi.org/10.1145/3292500.3330701.
43. T. Gaiddon, *et al.*, The harmonic-dominant signals in animal communication involve the use of new resonant frequencies. *Heliyon* **12**, e44424 (2026).
44. L. M. Roth, H. B. Hartman, Sound Production and Its Evolutionary Significance in the Blattaria. *Ann. Entomol. Soc. Am.* **60**, 740–752 (1967).
45. S. A. Hill, Sound generation in Mantis religiosa (Mantodea: Mantidae): stridulatory structures and acoustic signal. *J. Orthoptera Res.* **16**, 35–49 (2007).
46. K.-G. Heller, *Bioakustik der Europäischen Laubheuschrecken* (Verlag Josef Margraf, 1988).
47. D. A. Nickle, T. C. Carlysle, Morphology and function of female sound producing structures in ensiferan Orthoptera with special emphasis on the Phaneropterinae. *Int. J. Insect Morphol. Embryol.* **4**, 159–168 (1975).

48. S. Hugel, Panoploscelis scudderi Beier, 1950 and Gnathoclita vorax (Stoll, 1813): two katydids with unusual acoustic, reproductive and defense behaviors (Orthoptera, Pseudophyllinae). *Zoosystema* **40**, 327–340 (2019).
49. J. A. Cole, *et al.*, A revision of the shield-back katydid genus Neduba (Orthoptera: Tettigoniidae: Tettigoniinae: Nedubini). *Zootaxa* **4910**, 1–92 (2021).
50. F. Montealegre-Z, "Biomechanics of musical stridulation in katydids (Orthoptera: Ensifera: Tettigoniidae): an evolutionary approach." (2005).
51. D. D. Yager, Structure, development, and evolution of insect auditory systems. *Microsc. Res. Tech.* **47**, 380–400 (1999).
52. K. Kalmring, T. Sickmann, M. Jatho, R. Zhantiev, M. Grossbach, The auditory-vibratory sensory system of Polysarcus denticauda (Phaneropterinae, Tettigoniidae) 3. Physiology of the ventral cord neurons ascending to the head ganglia. *Journal of Experimental Zoology* **279**, 9–28 (1997).
53. J. Strauß, R. Lakes-Harlan, "Evolutionary and Developmental Adaptations of Tympanal Receptor Organs in Insects" in *Insect Hearing and Acoustic Communication*, B. Hedwig, Ed. (Springer Berlin Heidelberg, 2014), pp. 5–26.
54. S. R. Shaw, Detection of airborne sound by a cockroach "vibration detector": a possible missing link in insect auditory evolution. *Journal of Experimental Biology* **193**, 13–47 (1994).
55. K. G. Pearson, B. Hedwig, H. Wolf, Are the Hindwing Chordotonal Organs Elements of the Locust Flight Pattern Generator. *Journal of Experimental Biology* **144**, 235–255 (1989).
56. F. Mao, *et al.*, Fossils document evolutionary changes of jaw joint to mammalian middle ear. *Nature* **628**, 576–581 (2024).
57. F. Mao, *et al.*, Jurassic shuotheriids show earliest dental diversification of mammaliaforms. *Nature* **628**, 569–575 (2024).
58. P. G. Rodrigues, I. Ruf, C. L. Schultz, Digital Reconstruction of the Otic Region and Inner Ear of the Non-Mammalian Cynodont Brasilitherium riograndensis (Late Triassic, Brazil) and Its Relevance to the Evolution of the Mammalian Ear. *J. Mamm. Evol.* **20**, 291–307 (2013).

59. R. R. Hoy, “The Evolution of Hearing in Insects as an Adaptation to Predation from Bats” in *The Evolutionary Biology of Hearing*, D. B. Webster, R. R. Fay, A. N. Popper, Eds. (Springer-Verlag, 1992), pp. 115–129.
60. J. E. Yack, The structure and function of auditory chordotonal organs in insects. *Microsc. Res. Tech.* **63**, 315–337 (2004).
61. A. Y. Kawahara, *et al.*, Phylogenomics reveals the evolutionary timing and pattern of butterflies and moths. *Proceedings of the National Academy of Sciences* **116**, 22657–22663 (2019).
62. D. S. Jacobs, J. M. Ratcliffe, J. H. Fullard, Beware of bats, beware of birds: the auditory responses of eared moths to bat and bird predation. *Behavioral Ecology* **19**, 1333–1342 (2008).
63. J. P. Fournier, J. W. Dawson, A. Mikhail, J. E. Yack, If a bird flies in the forest, does an insect hear it? *Biol. Lett.* **9**, 20130319 (2013).
